# Genetic and Functional Modularity of *Hox* Activities in the Specification of Limb-Innervating Motor Neurons

**DOI:** 10.1371/journal.pgen.1003184

**Published:** 2013-01-24

**Authors:** Julie Lacombe, Olivia Hanley, Heekyung Jung, Polyxeni Philippidou, Gulsen Surmeli, Jonathan Grinstein, Jeremy S. Dasen

**Affiliations:** 1 Smilow Neuroscience Program, Department of Physiology and Neuroscience, Howard Hughes Medical Institute, NYU School of Medicine, New York, New York, United States of America; 2 Department of Biochemistry and Molecular Biophysics, Columbia University, New York, New York, United States of America; Stanford University School of Medicine, United States of America

## Abstract

A critical step in the assembly of the neural circuits that control tetrapod locomotion is the specification of the lateral motor column (LMC), a diverse motor neuron population targeting limb musculature. *Hox6* paralog group genes have been implicated as key determinants of LMC fate at forelimb levels of the spinal cord, through their ability to promote expression of the LMC-restricted genes *Foxp1* and *Raldh2* and to suppress thoracic fates through exclusion of *Hoxc9*. The specific roles and mechanisms of *Hox6* gene function in LMC neurons, however, are not known. We show that *Hox6* genes are critical for diverse facets of LMC identity and define motifs required for their in vivo specificities. Although *Hox6* genes are necessary for generating the appropriate number of LMC neurons, they are not absolutely required for the induction of forelimb LMC molecular determinants. In the absence of *Hox6* activity, LMC identity appears to be preserved through a diverse array of *Hox5–Hox8* paralogs, which are sufficient to reprogram thoracic motor neurons to an LMC fate. In contrast to the apparently permissive Hox inputs to early LMC gene programs, individual *Hox* genes, such as *Hoxc6*, have specific roles in promoting motor neuron pool diversity within the LMC. Dissection of motifs required for Hox in vivo specificities reveals that either cross-repressive interactions or cooperativity with Pbx cofactors are sufficient to induce LMC identity, with the N-terminus capable of promoting columnar, but not pool, identity when transferred to a heterologous homeodomain. These results indicate that Hox proteins orchestrate diverse aspects of cell fate specification through both the convergent regulation of gene programs regulated by many paralogs and also more restricted actions encoded through specificity determinants in the N-terminus.

## Author Summary

Coordinated motor behaviors—as complex as playing a musical instrument or as simple as walking—rely on the ability of motor neurons within the spinal cord to navigate towards and establish specific connections with muscles in the limbs. The establishment of connections between motor neurons and limb muscles is mediated through the actions of genes encoding Hox proteins, a large family of transcription factors conserved amongst all metazoans. However, the specific requirements for *Hox* genes in motor neuron specification and patterns of muscle connectivity are poorly understood. We have found that members of the *Hox6* gene paralog group (*Hoxa6*, *Hoxc6*, and *Hoxb6*) contribute to diverse aspects of motor neuron subtype differentiation. *Hox6* gene activity is required during two critical phases of motor neuron development: first as motor axons select a trajectory toward the forelimb and second as they choose specific muscles to innervate. At the molecular level, these two functions are encoded by distinct peptide domains within Hox proteins. This work indicates that Hox proteins execute their critical functions in motor neurons through intrinsic modules that confer distinct specificities and that these activities are central in the genetic network required for motor neuron differentiation.

## Introduction

The neural circuits that govern locomotor behaviors rely on the establishment of orderly sets of connections between motor neurons (MNs) and their peripheral and central synaptic targets. A critical and early step in the emergence of locomotor circuitry is the selection of specific muscle targets by a diverse array of MN subtypes. Three organizational features of MNs emerge during embryonic development that contributes to the specificity of their connections with target cells. First, MNs that project axons to common peripheral targets are organized into columns longitudinally arrayed along the rostrocaudal axis of the spinal cord [1], [2]. For example, MNs that project into the limb are contained within the lateral motor columns (LMCs), which are generated specifically at brachial and lumbar levels of the spinal cord. LMC neurons subsequently segregate into medial and lateral divisions, a program that dictates whether motor axons project into dorsal or ventral compartments of the limb mesenchyme [3], [4]. Finally, cells within each division further segregate into MN pools, each pool a cluster of stereotypically positioned MNs that innervates one of the ∼100 muscles in the limbs [2], [5]–[7]. MNs must therefore acquire a sufficient level of subtype diversity to ensure the appropriate muscle connectivity required for the emergence of coordinate locomotor behavior.

Within the developing spinal cord, Hox proteins exert central roles in the specification of MN columnar and pool subtypes [8], [9]. Nearly half of the 39 *Hox* genes are expressed by MNs, with subsets of related paralogs functioning at distinct levels of the MN differentiation pathway [10]. Three paralog groups, *Hox6*, *Hox9*, and *Hox10* genes have been implicated in the early columnar organization of MNs and contribute to the specificity of their initial projections into the periphery [11]–[13]. The actions of a much larger group of ∼20 *Hox* genes contribute to the specification of MN pools, in part, through the induction of intermediate transcription factors [10], [14]–[16]. During these programs of MN diversification, Hox proteins mediate both the selective activation of downstream targets and the exclusion of other determinants through mutual cross repression, two distinct activities that appear to be intrinsic to Hox proteins [10], [11]. Despite significant progress towards defining roles for Hox proteins in MNs, the mechanisms by which they control diverse features of MN subtype identity are largely unknown.

Studies in *Drosophila* indicate two key mechanisms through which Hox proteins regulate target genes [17]. The first involves the selection of DNA target sites. Hox proteins typically display low affinity for DNA in vitro, with high fidelity binding requiring cooperative interactions with the TALE-domain containing homeodomain factors extradenticle and homothorax (Pbx and Meis proteins in vertebrates) [18]. While TALE-domain protein interactions increase the affinity and selectivity of Hox proteins for DNA, they have only a subtle influence on the specificity of site selection in vitro, particularly amongst Hox proteins expressed in more caudal regions of the embryo [19]–[21]. Recent evidence, however, suggests that in vivo specificity can be achieved by sequences N-terminal to the homeodomain, which mediate contacts with the minor groove at target sites [22], [23]. Once bound to a target gene, the activities of Hox/Pbx complexes can be further modulated through the actions of ancillary transcription factors that typically bind in proximity to Hox targets [24], [25]. In this mode of action, a Hox protein may not depend as much on DNA site selection for specificity, but rather on how it interacts with factors it engages at a target sequence.

Some insights into the mechanisms by which Hox proteins regulate target genes in MNs have emerged through analysis of mice mutant for a single thoracically expressed *Hox* gene, *Hoxc9*. Hoxc9 is required for the appearance of thoracic-level MN columnar subtypes including preganglionic column (PGC) and hypaxial motor column (HMC) neurons [26]. A critical aspect of Hoxc9 function is to establish the boundary between thoracic and forelimb-level MN populations through cross repression, as in the absence of *Hoxc9* all brachial *Hox* genes are derepressed at thoracic levels, and MNs acquire an LMC fate. This broad repressive activity appears to be mediated by direct interactions of Hoxc9 with multiple sites in the *HoxA* and *HoxC* loci. Genome-wide analysis of Hoxc9 binding revealed a consensus binding motif which matches a high affinity Hox/Pbx site (TGATTTAT) identified by several groups through in vitro site selection [19], [20]. This sequence engages a wide range of Hox paralogs, raising the issue of how the in vivo specificities of Hox proteins in MNs are achieved if they are not dependent on the recognition of specific genomic target sites.

The problem of Hox specificity in MNs is particularly relevant at limb levels of the spinal cord, where individual neurons express multiple Hox proteins at the time of their differentiation [10], [11], [27]. In this context Hox proteins appear to contribute to both gene programs common to all LMC neurons as well as more restricted actions necessary for diversification of LMC neurons into MN pools. At limb levels of the spinal cord the actions of *Hox6* and *Hox10* genes have been implicated in the initiation of the LMC program at brachial and lumbar levels respectively, through activation of the gene encoding the transcription factor FoxP1 [28]. FoxP1 is subsequently required for the expression of the gene encoding the retinoic acid synthesizing enzyme *Raldh2* [28], [29]. This MN-derived source of retinoids is necessary for the Lim homeodomain protein-mediated segregation of the LMC into medial and lateral divisions [30], [31]. Thus the deployment of the LMC program at forelimb and hindlimb levels is mediated by two distinct sets of Hox paralogs that activate a common set of downstream pathways required for MN columnar and divisional specification.

While Hox proteins seem to be critical for LMC specification, it is less clear how they contribute to MN pool diversity. At brachial levels the LMC is broadly divided into rostral and caudal domains by expression of *Hox5* genes (*Hoxa5* and *Hoxc5*) and *Hoxc8*, respectively; and the actions of these *Hox* genes are necessary for delineating the rostrocaudal position of MN pools [10]. Within a given segment a repression-based network of *Hox4–Hox8* proteins are thought to promote the intrasegmental diversity of MNs, by defining specific molecular codes for each pool subtype. For example, misexpression studies in chick have provided evidence that Hoxc6 is selectively required for the intrasegmental differentiation of pools within the caudal (Hoxc8+) half of the LMC [10]. Thus the same *Hox6* paralog group that determines the early columnar identity of forelimb-innervating MNs contains members that promote motor pool fates.

In this study we sought to address several unresolved issues concerning the function and specificity of *Hox6* genes during MN columnar and pool specification programs. First, what are the specific contributions of the three murine *Hox6* genes to MN fate specification? Second, to what extent are the diverse activities of a Hox protein unique, or are they shared amongst gene paralogs within a cluster? Third, are there motifs intrinsic to Hox proteins that subfunctionalize in vivo specificities? To address these questions we analyzed mice in which all *Hox6* genes are mutated, as well as employed an in vivo approach to dissect functional domains required for Hox specificity in MNs. We find that although LMC specification is retained in mice lacking *Hox6* genes, *Hoxc6* has a specific role in promoting MN pool identity and appropriate patterns of limb connectivity. The preservation of LMC fate in *Hox6* mutants appears to be mediated by a diverse group of *Hox5–Hox8* genes expressed at brachial levels. Dissection of a single Hox protein reveals in vivo specificity relies on motifs that ensure deployment of programs common to all LMC neurons, as well as distinct modules that contribute to MN pool identity.

## Results

### Impairment of Lateral Motor Column specification in *Hox6* mutant mice

**Figure 1:**
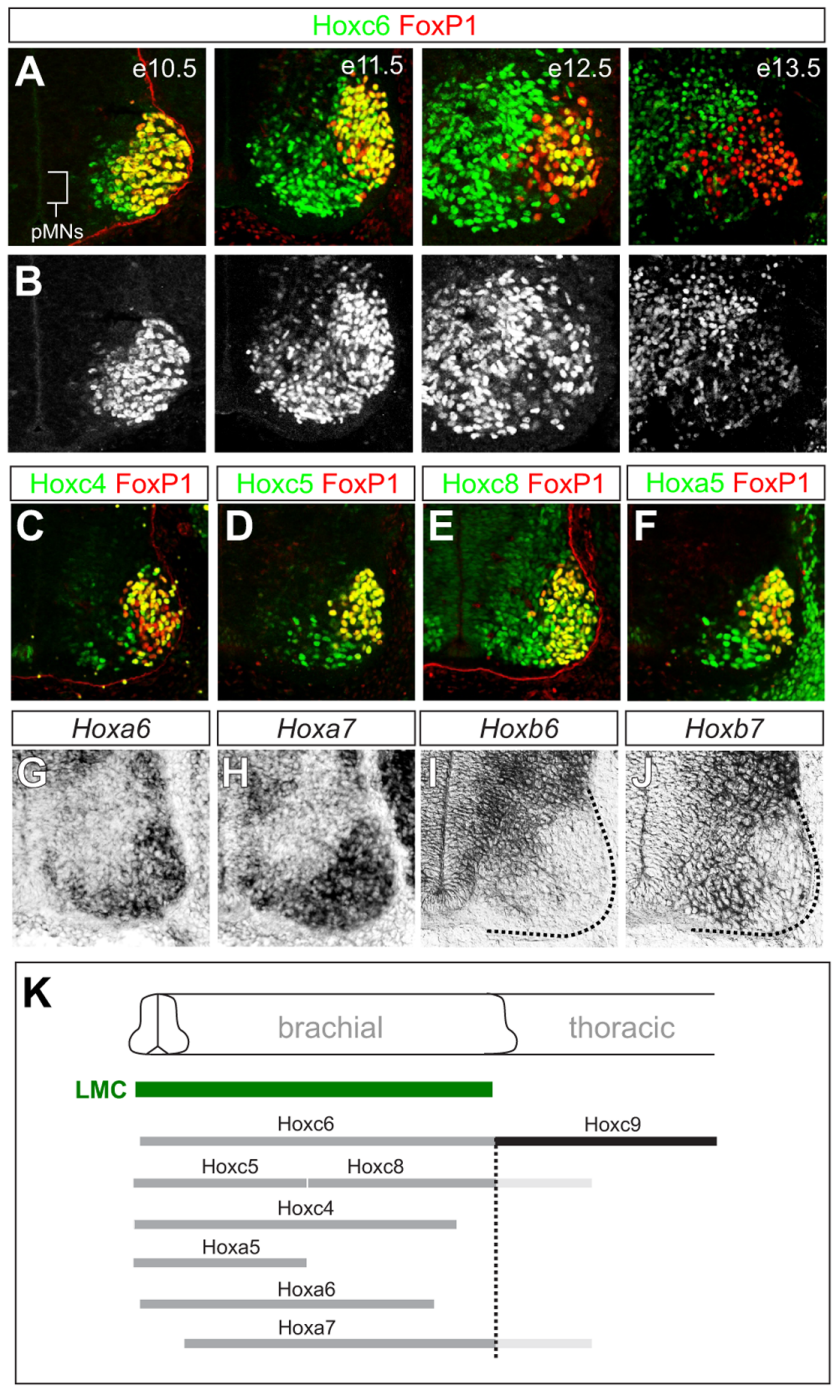
Dynamic expression of brachial *Hox* genes in the spinal cord. (A) Expression of Hoxc6 and FoxP1 in LMC neurons between e10.5 and e13.5. Images show ventral quadrant of the spinal cord and LMC neurons are identified by FoxP1 expression. Hoxc6 protein is detected in postmitotic MNs but not in MN progenitors (pMNs). At e10.5 Hoxc6 is expressed by FoxP1+ LMC neurons but by e12.5 is attenuated in most LMC neurons. (B) Grayscale images showing progressive decline in Hoxc6 protein expression from LMC neurons. (C–J) Expression of *Hox4–Hox8* paralogs at brachial levels of the spinal cord. *Hoxb6* and *Hoxb7* are expressed in pMNs. (K) Summary of Hox expression patterns. Hoxc4, Hoxa5, Hoxc6 expression domains extend further rostrally in the spinal cord and are not shown. Hoxa7 and Hoxc8 expression also extends into thoracic levels, indicated in light grey.

Studies in chick have implicated *Hox6* genes in the specification of LMC neurons at brachial levels of the spinal cord. Two *Hox6* genes, *Hoxa6* and *Hoxc6*, are selectively expressed by brachial MNs in chick, and can convert HMC and PGC neurons to an LMC fate when misexpressed at thoracic levels [11]. Whether *Hox6* activities are absolutely required for LMC specification in mice is not known. To begin to answer this question we first analyzed the expression of *Hox6* paralogs (*Hoxa6*, *Hoxb6*, and *Hoxc6*) at brachial levels near the time of LMC differentiation at embryonic day (e) 11.5. *Hoxa6* and *Hoxc6* are expressed throughout the brachial LMC, while *Hoxb6* is expressed by MN progenitors (Figure 1A, 1B, 1G, 1I). *Hox6* genes also displayed temporally dynamic patterns; after e11.5 *Hoxa6* expression was only weakly detected in the spinal cord, while Hoxc6 was attenuated in subsets of LMC neurons by e12.5, and downregulated in most LMC neurons by e13.5 (Figure 1A, 1B and data not shown).

**Figure 2:**
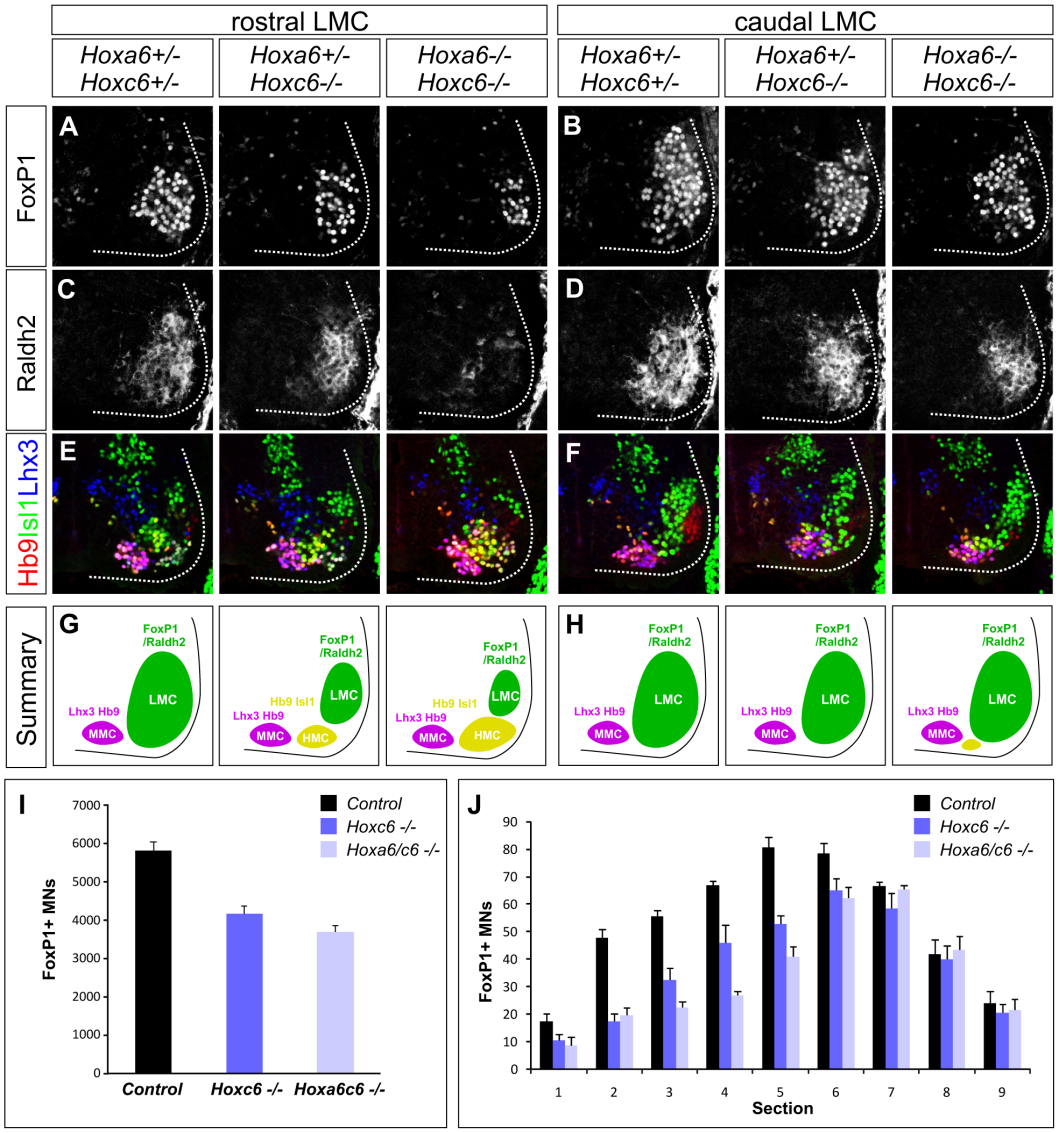
Analysis of motor neuron columnar specification in *Hox6* mutant mice. (A–D) Loss of LMC neurons at brachial levels of the spinal cord in *Hoxa6/Hoxc6* mutants at e12.5. LMC neurons are defined by FoxP1 and Raldh2 expression. The most pronounced losses are observed in rostral brachial regions (A, C), where the number of FoxP1 MNs is reduced and Raldh2 is only weakly detected in *Hoxa6/Hoxc6* double mutants. (E–F) Increase in the number of Hb9+, Isl1+, Lhx3− HMC neurons in *Hox6* mutants. Rostral brachial spinal cord also normally contains a small MN population that coexpresses Hb9 and Isl1, but for simplicity is not shown. The number of Lhx3+ MMC neurons is unchanged. (G–H) Summary of MN columnar defects in *Hoxa6/c6* mutants. (I) Quantification of total number of LMC neurons in *Hox6* mutants. Numbers are extrapolated from serial sections and are based on FoxP1 MN counts. Controls are averaged from *Hoxa6*−/− *Hoxc6+/+*, *Hoxa6+/−Hoxc6+/−*, and *Hoxa6−/−, Hoxc6+/−* embryos as the number of LMC neurons in these alleles was similar to wildtype embryos (see Figure S3). (J) Quantification of LMC loss in serial sections of *Hoxa6/c6* mutants. Sections numbered 1–9 represent progression from rostral to caudal levels of the LMC.

To assess the function of *Hox6* genes in LMC neurons we generated and analyzed mice containing various combinations of *Hox6* mutant alleles [32], [33]. Single and combined mutation of *Hoxa6* and *Hoxc6* had no effect on general features of MN identity, as assessed by the presence of the early MN determinants Hb9, Islet1/2 and Lhx3 (Figure 2E, 2F; Figure S1A). In addition *Hoxa6* was not upregulated in *Hoxc6* mutants, nor was *Hoxb6* upregulated in MNs of *Hoxa6/c6* mutants, and the normal patterns of brachially expressed HoxA and HoxC proteins were maintained (Figure S1B, S1C and data not shown). Moreover, the thoracic *Hoxc9* gene was not noticeably derepressed at brachial levels in *Hox6* mutants (Figure S2), likely due to compensation by other *Hox* paralog groups (see below). *Hox6* genes are therefore not required for the generation of MNs as a class or in maintaining Hox expression patterns.

We next determined the profile of LMC determinants in *Hoxa6/c6* mutants. We assessed the expression of *Foxp1* and *Raldh2*, two genes that are induced downstream of Hox proteins [28]. In *Hoxa6*−/−*Hoxc6+/+*, *Hoxa6+/−Hoxc6+/−*, and *Hoxa6−/− Hoxc6+/−* embryos the number of LMC neurons was similar to wildtype embryos, while both *Hoxc6−/−* and *Hoxa6−/−Hoxc6−/−* mutants displayed significant LMC losses (Figure 2A–2D, 2I–2J; Figure S3A). To quantify the reduction of LMC neurons in *Hoxc6−/−* and *Hoxa6−/−Hoxc6−/−* embryos, we performed serial sectioning on e12.5 embryos and determined the total number of FoxP1+ LMC neurons averaged from n>3 mutants and control littermates. This analysis revealed a 28% loss of LMC neurons in *Hoxc6* mutants, and a 37% loss in *Hoxa6/Hoxc6* double mutants (Figure 2I–2J). The loss of LMC neurons was particularly prominent in the rostral half of the LMC (Hoxa5/c5+ region), where we observed a 41% decrease in FoxP1+ MNs in *Hoxc6* mutants and a 56% decrease in *Hoxa6/c6* double mutants (Figure 2A, 2C). In addition mutation of *Hoxa6/c6* had a more severe impact on Raldh2 expression, with a near complete absence of expression in the rostral brachial spinal cord, possibly due to an attenuation in FoxP1 expression levels in the remaining MNs (Figure S3B). Similar defects in LMC specification were observed at e10.5 and e11.5, indicating they are present at the time of LMC generation (Figure S3B–S3D). *Hoxa6* and *Hoxc6* are therefore necessary for the appearance of the normal number of LMC neurons.

We next determined the fate of the LMC neurons that are lost in *Hoxa6/c6* mutant mice. Analysis of *Foxp1* mutants suggests that in the absence of a Hox-programmed LMC identity, MNs remain in the “default” fate of the hypaxial motor column (HMC) subtype, a motor neuron column normally present at thoracic levels [28], [29]. Consistent with this idea, we find an increase in the number of MNs with an HMC character, defined by high levels of Hb9 and Isl1 coexpression (Figure 2E–2H, Figure S1A). In contrast the number of Lhx3+ Hb9+ MMC neurons, a Hox-independent columnar subtype present at all levels of the spinal cord, was unchanged (Figure 2E–2H). These observations demonstrate that in the absence of *Hoxa6/Hoxc6*, MNs that fail to acquire an LMC fate revert to an HMC-like identity.

Because expression of *Hoxb6* in MN progenitors could account for the maintenance of LMC identity in *Hoxa6/Hoxc6* mutants we also analyzed mice in which all three murine *Hox6* alleles are deleted. We found that in *Hoxa6/Hoxb6/Hoxc6* triple mutants FoxP1+/Raldh2+ MNs were present, and LMC numbers were grossly similar to *Hoxa6/Hoxc6* double mutants (Figure S4). As in *Hoxa6/Hoxc6* mutants, the LMC loss was most prevalent at more rostral brachial levels, while caudal brachial LMC MNs were less affected (Figure S4). Thus *Hox6* genes are necessary for appropriate LMC numbers, but are not absolutely required for the activation of LMC molecular determinants in brachial spinal cord.

### Hox5–Hox8 proteins determine common and distinct features of LMC MNs

The perseverance of LMC identity in *Hox6* mutants raises the question of whether other *Hox* paralogs might contribute to their specification. To address this question we began by analyzing the expression patterns of additional *Hox* genes at brachial levels. In chick spinal cord several genes belonging to the *Hox4–Hox8* paralog groups are expressed by brachial LMC neurons. We therefore determined the expression patterns of *Hox4–Hox8* genes in mouse at e11.5 with reference to the brachial LMC. This analysis revealed patterns of *Hox4–Hox8* paralog expression that were similar to patterns in chick [10], with *HoxA* and *HoxC* cluster genes (*Hoxa5, Hoxa6, Hoxa7, Hoxc4, Hoxc5, Hoxc6, Hoxc8*) the most prominently expressed by brachial MNs (Figure 1A–1K). These observations indicate that Hox patterns are largely conserved between mouse and chick, with multiple *Hox* genes expressed by brachial LMC neurons at the time of their differentiation.

**Figure 3:**
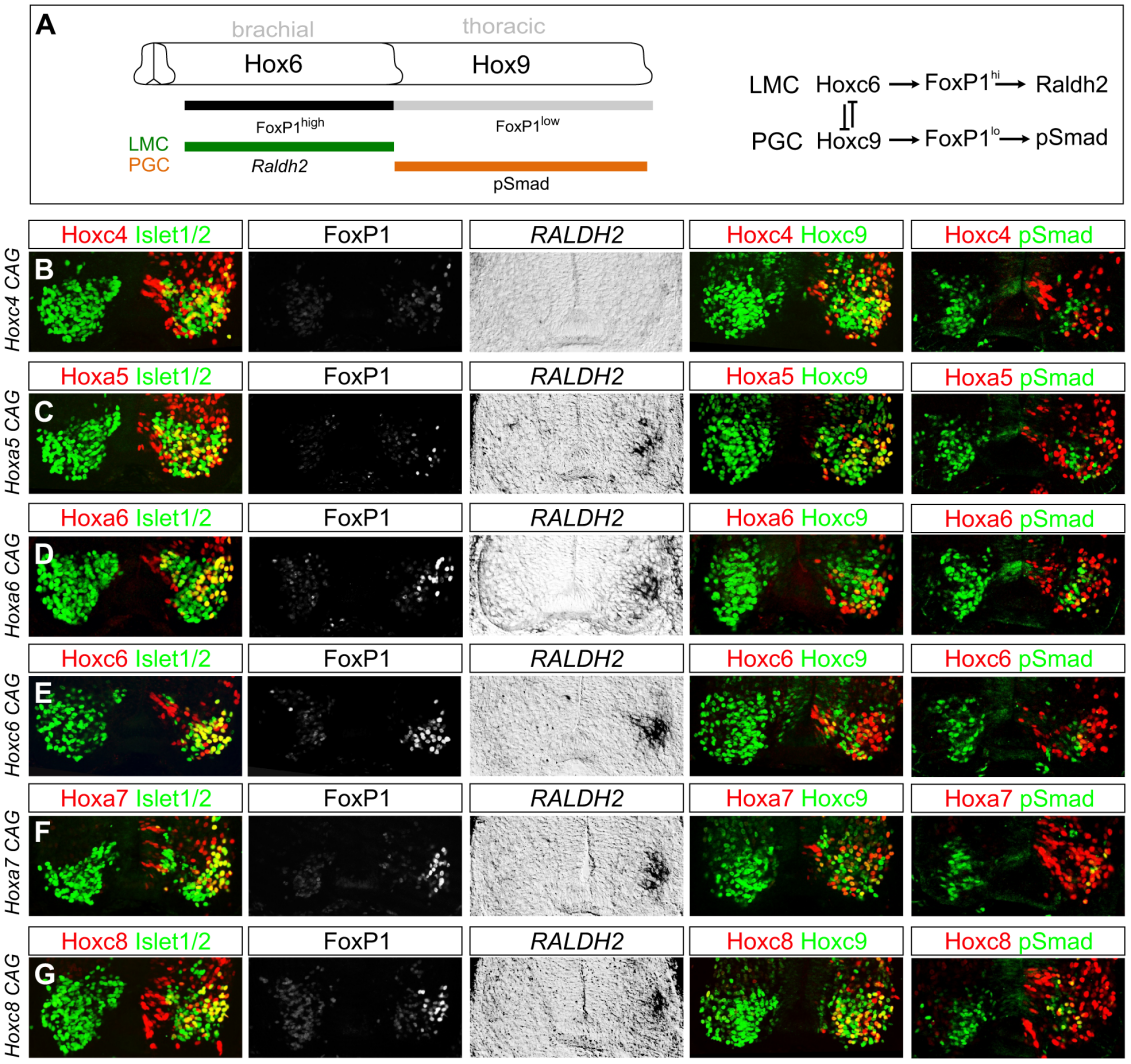
Multiple *Hox* proteins can program LMC identity at thoracic levels. (A) Schematic of Hox patterns and Hox-dependent MN columnar subtypes at brachial and thoracic levels. Lateral motor column (LMC) neurons express high FoxP1 levels and Raldh2. Preganglionic column (PGC) neurons express low FoxP1 levels and pSmad. Model on right shows Hox interactions specifying LMC and PGC MN subtypes. (B–G) Effects of misexpression of *Hox4–Hox8* genes at thoracic levels. Electroporated motor neurons are indicated by Hox protein+Isl1/2 costaining. (B) Hoxc4 fails to induce *Foxp1* or *Raldh2* expression and does not repress *Hoxc9*. (C) Hoxa5 induces LMC identity, blocks pSmad, but fails to repress *Hoxc9*. (D–E) Hoxa6 and Hoxc6 induce LMC MNs and effectively extinguish *Hoxc9* and pSmad. (F–G) Hoxa7 and Hoxc8 induce LMC fate and display attenuated capacity to repress *Hoxc9*. Hoxa7 and Hoxc8 electroporations are shown at caudal thoracic levels, where endogenous expression is minimal. Embryos were electroporated at HH st13–14 and analyzed two days later.

To determine the influence of brachially expressed *Hox4–Hox8* genes on MN differentiation we used chick neural tube electroporation to assess the effects of misexpression at thoracic levels. Since Hox overexpression could lead to neomorphic effects, we optimized plasmid concentrations in electroporations to be qualitatively similar to levels found in the endogenous brachial domain in chick (Figure S5A, S5B). We compared the effects of thoracic Hox misexpression to that of Hoxa6 and Hoxc6, which have been shown to induce LMC identity at thoracic levels (Figure 3A) [11], [28]. Consistent with previous observations, Hoxc6 and Hoxa6 were similar in their capacity to induce expression of markers of LMC identity including high levels of FoxP1 and *Raldh2*, and to cell autonomously abolish expression of thoracic determinants of PGC fate, including *Hoxc9* and phospho (p) Smad1/5/8 (Figure 3D, 3E).

We next tested the effects of thoracic misexpression of representative genes from the *Hox4*, *Hox5*, *Hox7*, and *Hox8* paralog groups. We observed that thoracic misexpression of either Hoxa5, Hoxa7 or Hoxc8 could redirect thoracic MN fate towards a brachial LMC identity as assessed by induction of *Foxp1* and *Raldh2* and loss of pSmad expression (Figure 3C, 3F, 3G). In contrast, although Hoxc4 is expressed by most LMC neurons, thoracic misexpression of Hoxc4 failed to induce high FoxP1 or *Raldh2* (Figure 3B). These observations indicate that many, but not all, brachially expressed *Hox* genes can promote an LMC character at thoracic levels.

We additionally found that the capacity of different Hox proteins to specify aspects of LMC identity varies depending on the Hox protein. While 64% of thoracic MNs expressing Hox6 proteins acquired an LMC identity, Hoxa5 only induced LMC fate in 41% of electroporated MNs (Figure S5C). Hox proteins thus differ in the extent to which they can generate LMC neurons. The reduced potential of Hoxa5 to promote LMC fate likely accounts for the more pronounced decline in LMC numbers at rostral brachial levels in *Hox6* mutants, as this region lacks the more potent LMC inducers, Hoxa7 and Hoxc8. In addition Hoxc8, Hoxa7, and Hoxa5 were less effective than Hox6 proteins in extinguishing *Hoxc9* expression, and many of these Hox-induced LMC motor neurons continued to express Hoxc9 (Figure 3C, 3F, 3G). Together these observations indicate that multiple Hox proteins can specify features common to all LMC neurons, while individual Hox proteins diverge with respect to LMC promoting efficacies and cross-repressive activities.

### Hoxc6 has a selective function in motor neuron pool differentiation

While multiple *Hox* genes appear to converge in regulating early programs of brachial LMC differentiation, it is possible that they have distinct roles during motor neuron pool diversification within the LMC. Within the *Hox6* paralog group, *Hoxc6* has been implicated in the specification of MN pools innervating specific muscles in the chick embryo, independent of its function in promoting LMC identity [10]. We therefore determined whether *Hoxc6* has an obligate role in MN pool differentiation.

**Figure 4:**
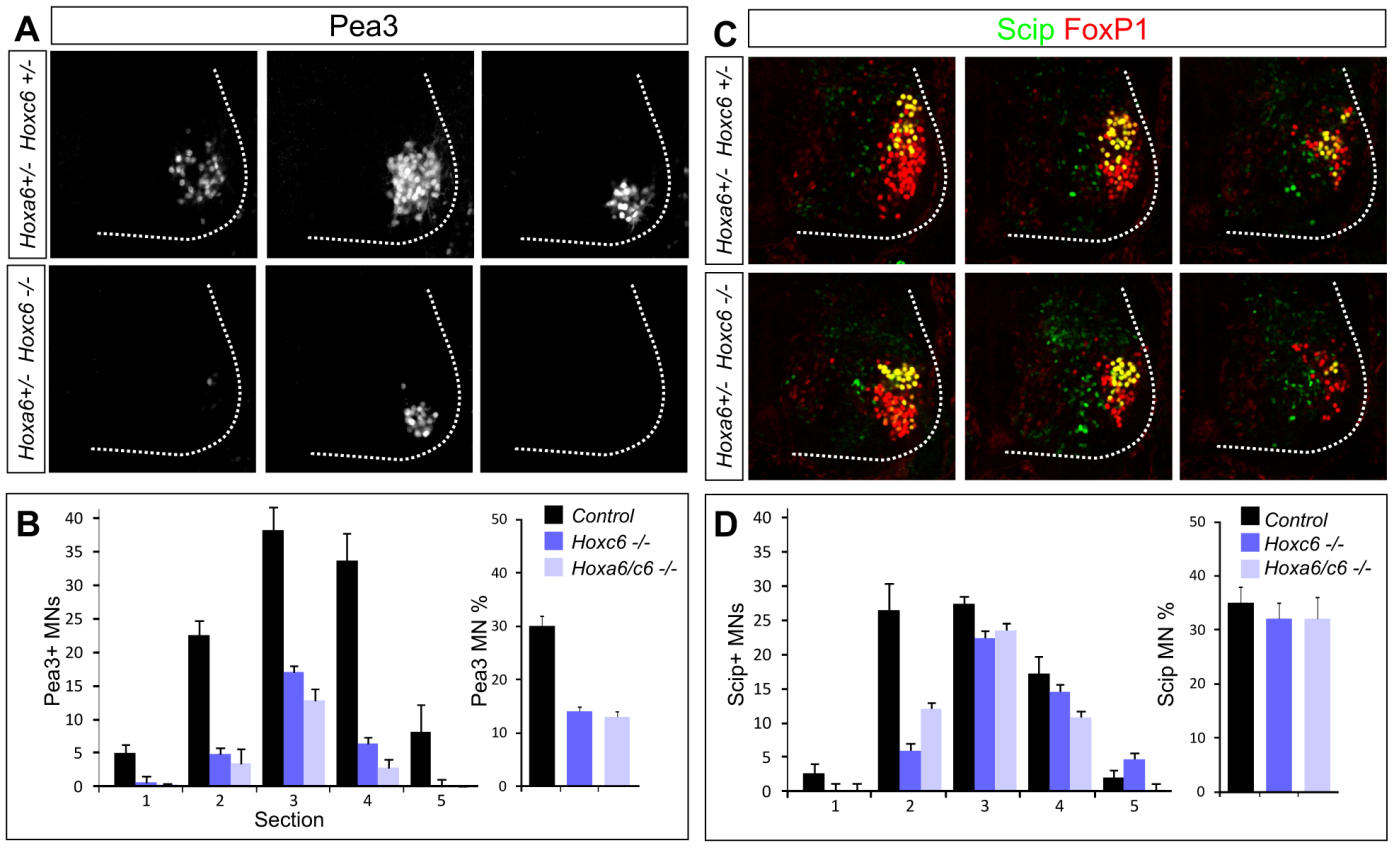
*Hoxc6* is required for the specification of the Pea3 motor neuron pool. (A) Serial sections from the caudal half of the LMC in *Hoxc6* mutants showing loss of the Pea3+ pool at e12.5. Rostral to caudal sections are shown from left to right. Images are taken from a level similar to sections 1, 3, and 5 in panel D. Results are similar between *Hoxc6* and *Hoxa6/Hoxc6* double mutant sections. (B) Quantification of the loss of Pea3+ MNs in *Hox6* mutants. Graph on left shows average Pea3+ MN number per section. Graph on right shows Pea3+ MN number as a percentage of the total LMC. (C) Expression of Scip+ LMC neurons in *Hoxc6* mutants. Images are taken from a level similar to sections 3, 4, and 5 in panel D. (D) Quantification of Scip+ MNs in *Hox6* mutants. Graph on left shows Scip+ MN number per section. Graph on right shows Scip MN number as a percentage of the total LMC.

Within the caudal half of the brachial LMC, some MN pools can be defined by expression of the Ets protein Pea3 as well as the POU domain protein Scip [26], [34], [35]. In the caudal (Hoxc8+) half of the LMC, Hoxc6 has been argued to promote the specification of the Pea3+ pool and restrict expression of Scip [10]. We therefore assessed the specification of these pools in *Hox6* mutants. While the number of FoxP1+ LMC neurons generated at caudal brachial levels was not significantly reduced in *Hoxa6/c6* mutants, we observed a significant defect in motor neuron pool differentiation. In both *Hoxc6* and *Hoxa6/c6* double mutants the number of Pea3+ MNs was markedly reduced, while the Scip+ pool was relatively spared (Figure 4A–4D, Figure S6E). Because LMC neuron numbers are reduced overall in *Hoxc6* mutants we quantified the number of Pea3+ and Scip+ MNs as a percentage of the total number of LMC neurons generated. Within the rostrocaudal limits of the pool, Pea3 MNs account for 30% of all LMC neurons; and in *Hoxc6* mutants this number was reduced to 12% (Figure 4B). Scip+ MNs account for 35% of LMC neurons within its limits, and in *Hoxc6* mutants, this number was 32% (Figure 4D). Thus *Hoxc6* is selectively required for the normal appearance of the Pea3 pool, independent of its role in LMC specification, but only has a minor contribution to Scip+ LMC neurons.

### Loss of *Hoxc6* leads to innervation defects in the forelimb

**Figure 5:**
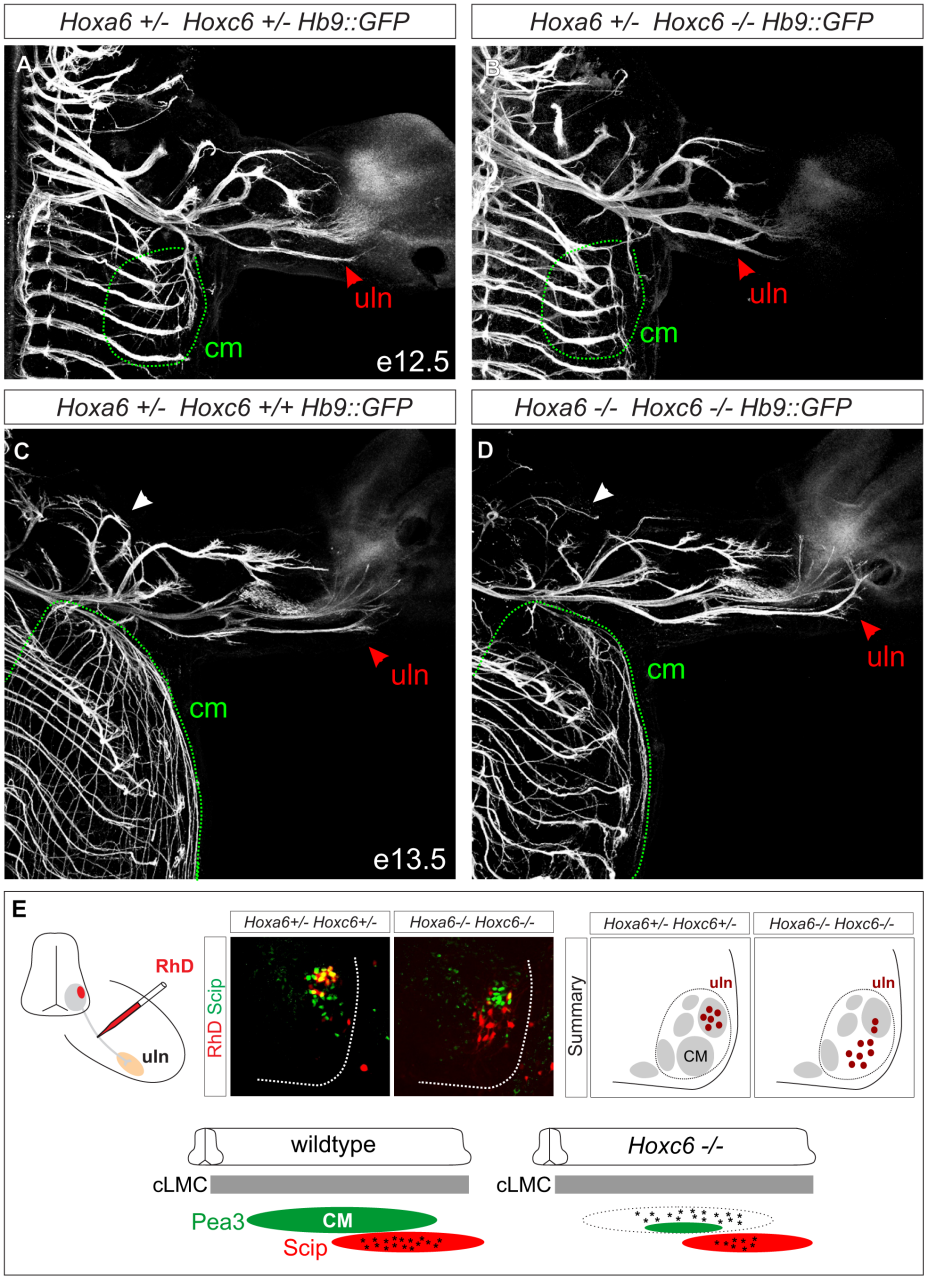
Limb innervation defects in *Hoxc6* mutants. (A–D) Whole mount GFP staining in *Hb9::GFP, Hoxc6* mutant and control mice. At both e12.5 and e13.5 there is a severe reduction in the innervation of the cutaneous maximus (cm) muscle (outlined with dashed green line). At e12.5 *Hoxc6* mutants are characterized by an abnormal bifurcation at the distal end of the ulnar (uln) nerve, and at e13.5 there is an atypical misdirection of axons towards the medial portion of the paw (red arrowheads in A–D). Musculocutaneous nerve is indicated by white arrowheads in panels C and D. Similar results were obtained in n = 3 control and *Hoxc6* mutants at both ages. (E) Retrograde labeling from the ulnar nerve in *Hoxc6* mutants. In *Hoxc6* mutants, many Scip− MNs are labeled with RhD. In addition there is a reduction in the number of Scip+ RhD+ labeled MNs in the *Hoxc6* mutant. Section shown for the *Hoxc6* mutant is rostral to the central portion of the Scip+ LMC pool, at a segmental level occupied predominantly by former Pea3+ MNs (See also Figure S7). Schematic summarizes the distribution of labeled MNs after tracer injection into the ulnar nerve in caudal (c) LMC neurons.

In mice, Pea3 is expressed by MNs that target the cutaneous maximus (CM) muscle, whereas Scip+ MN pools project along the median and ulnar nerve [26], [35]. To further assess the impact of loss of *Hoxc6* on MN development, we bred *Hox6* mutant mice to a line in which all MNs are labeled with GFP (*Hb9::GFP* mice) [36], [37] and analyzed motor axon projections in the limb. In *Pea3* mutants, motor axons project to the CM but fail to branch and arborize the muscle [35]. Consistent with a loss of the Pea3+ MN pool there was a drastic reduction in the arborization of the CM in *Hoxc6* mutants (Figure 5A–5D, Figure S6A–S6D). In addition distal branches of the musculocutaneous nerve were poorly formed (Figure 5A–5D, Figure S6A–S6D), suggesting that *Hoxc6* may have roles in the specification of additional pools that cannot yet be defined by unique molecular markers.

Interestingly, projections along the ulnar nerve were not reduced, but instead displayed supernumerary branches at the distal end, which were atypically directed towards the paw at e12.5 and e13.5 (Figure 5B, 5D; Figure S6B, S6D). We considered the possibility that the ulnar nerve might receive innervation from LMC neurons that have lost Pea3. To test this we injected Rhodamine (RhD) dextran tracers into the ulnar nerve and assessed the molecular identity of retrogradely labeled MNs. In control animals all RhD-labeled MNs expressed Scip, while in *Hoxc6* mutants many RhD+ Scip− neurons were observed (Figure 5E, Figure S7A–S7C). Many of these RhD+ Scip− MNs were located more rostral to the Scip pool, occupying a position where Pea3+ MNs would normally reside (Figure S7). Thus *Hoxc6* is required for the specification of Pea3+ CM MNs, and in the absence of this program many motor axons appear to acquire the projection characteristics of ulnar MNs.

### Columnar and pool specification are mediated by distinct Hox activities

Collectively, our findings suggest that while multiple *Hox* genes share a common function in promoting LMC fates they diverge with respect to MN pool specification. To understand the basis for the differential activities of Hox proteins in MNs we searched for intrinsic domains that contribute to their MN-specific activities in vivo. We decided to focus on Hoxc6 for this analysis as it is initially expressed by the majority of brachial LMC neurons, its activities are required for normal LMC generation, the specification of the Pea3+ pool, and it can extinguish *Hoxc9* through its repressive functions.

**Figure 6:**
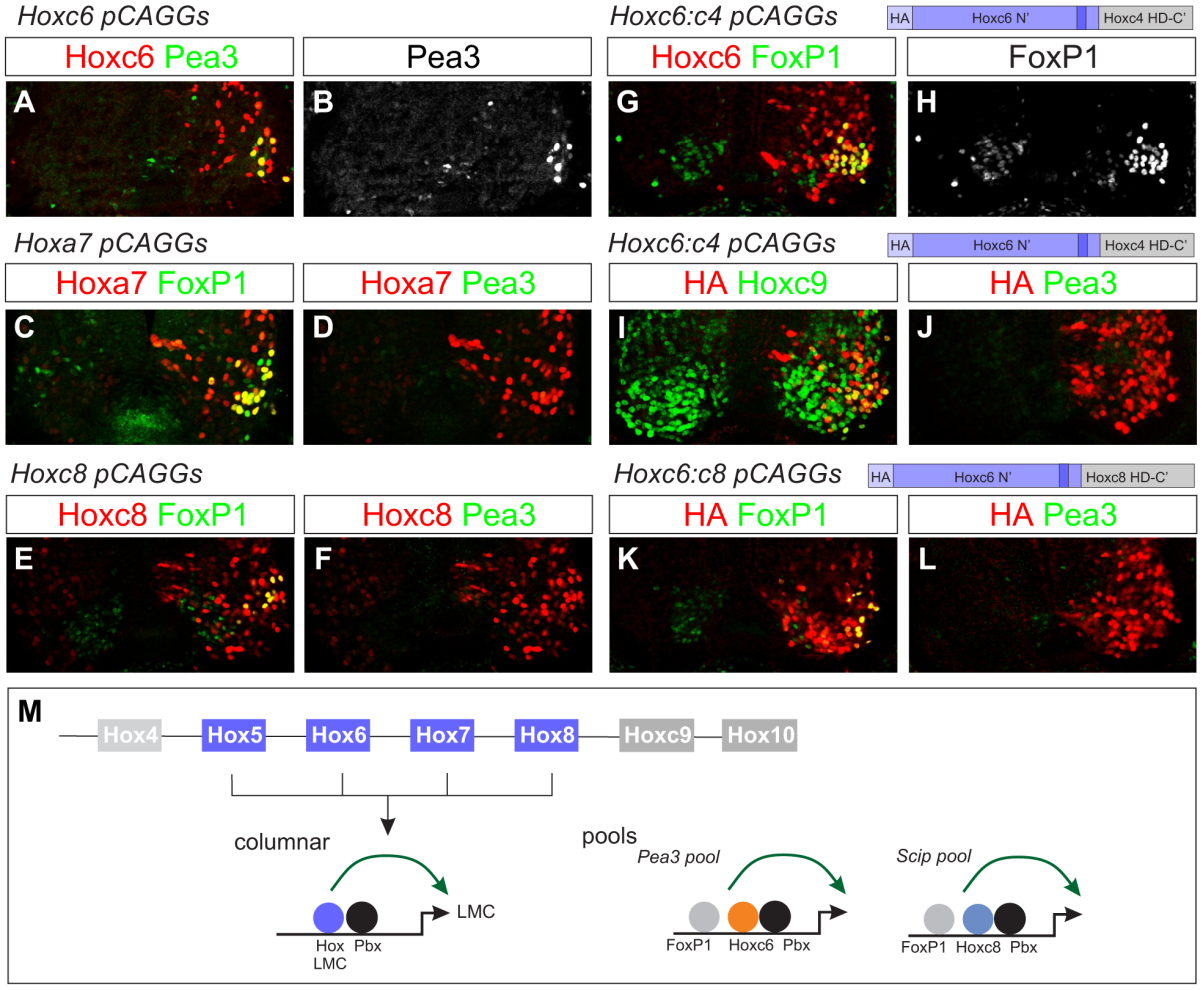
Effects of native and chimeric Hox proteins on columnar and pool specification. (A–B) Electroporation of Hoxc6 induces *Pea3* expression at thoracic levels. (C–F) Expression of Hoxa7 and Hoxc8 at thoracic levels induces *Foxp1* but not *Pea3* expression. Similar results were obtained with Hoxa5 (data not shown). (G–H) A chimera of the Hoxc6 N-terminus (including the YPWM motif) and the Hoxc4 homeodomain (HD) induces high levels of *Foxp1* expression. (I–J) The Hoxc6:c4 chimera fails to induce *Pea3* expression at thoracic levels and shows an attenuated capacity to repress Hoxc9. (K–L) A chimera of the Hoxc6 N-terminus to the homeodomain of Hoxc8 induces LMC MNs, but fails to induce *Pea3* expression. (M) Model for Hox interactions in MN subtype specification. During LMC specification multiple Hox paralogs converge on the regulation of pan-LMC genes such as FoxP1 and Raldh2. In motor neuron pools more specific Hox activities are employed.

We first asked whether the capacity of Hoxc6 to promote the identity of the Pea3 pool reflects a specific activity of this particular *Hox* gene. Consistent with a restricted role in promoting Pea3+ MN fates, we find that Hoxc6 can induce *Pea3* in a subset of the ectopic FoxP1+ LMC neurons generated after thoracic misexpression (Figure 6A, 6B). In contrast, thoracic expression of Hoxa5, Hoxa6, Hoxa7, and Hoxc8 failed to induce expression of *Pea3* within the ectopic LMC population (Figure 6C–6F and data not shown). Thoracic Hoxc6 expression is therefore sufficient to promote both columnar and pool fates at thoracic levels, and its Pea3 pool promoting activity appears to be unique to Hoxc6.

Next we defined regions that contribute to the ability of Hoxc6 to induce columnar and pool fates. We first tested the activities of chimeras between the N-terminus of Hoxc6 (containing all amino acids up to the homeodomain) and the homeodomains of heterologous Hox proteins. Fusion of the Hoxc6 N-terminus to the homeodomain (HD) of the “LMC-neutral” Hox protein, Hoxc4 (Figure 3B), activated high levels of *Foxp1* at thoracic levels (Figure 6G, 6H). Thus the LMC inducing actions of Hoxc6 can be transferred to a Hox protein that normally cannot induce LMC fates. However, this chimera failed to induce expression of the pool marker *Pea3*, and many MNs continued to express Hoxc9 (Figure 6I, 6J). Similarly, fusion of the Hoxc6 N-terminus to the HD of Hoxc8 induced an LMC fate, but failed to promote *Pea3* expression (Figure 6K, 6L). These observations indicate that Hox proteins rely on the activities of both the N-terminal and HD sequences, with the N-terminal region sufficient for LMC induction and the N-terminal+HD controlling aspects of MN pool specification. Thus while early LMC programs are initiated through activities presumably common to many Hox proteins, pool-restricted actions require a coherent N-terminal and HD region (Figure 6M).

### Characterization of DNA binding activities of Hoxc6 mutant derivatives

Hox proteins are known to contain peptide motifs that confer activation and repression of target genes independent of the homeodomain [38]–[40], although how these activities contribute to MN columnar and pool identities are unclear. Based on our analysis of Hox chimeras we next asked whether the actions of Hoxc6 in LMC specification are mediated through modules in the N-terminus. Specifically we sought to define whether there are specific domains that determine how Hoxc6 promotes LMC fate, the specification of the Pea3 pool, and represses *Hoxc9*.

**Figure 7:**
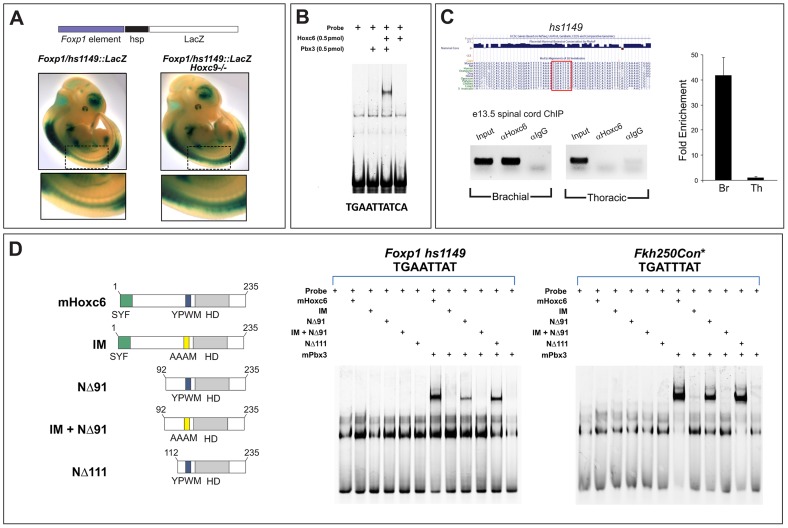
Analysis of binding properties of Hoxc6 mutant derivatives. (A) Analysis of a *Foxp1* enhancer transgene (*hs1149::lacZ*) expression in the spinal cord. This enhancer targets high levels of *lacZ* expression at limb-levels of the spinal cord, but low levels thoracically. Expression is upregulated in *Hoxc9* mutants indicating it is under Hox control. Further analysis of this line however revealed that this enhancer targets a broad range of cell types (data not shown), suggesting that other elements are required to achieve MN expression and specificity. (B) Gel mobility shift assays with Hoxc6 and Pbx3 on a putative Hox site in *hs1149*. (C) ChIP analysis of Hoxc6 binding to the *hs1149* region in mouse spinal cord. (D) Binding of Hoxc6 mutant derivatives to *hs1149* and an optimized Hox binding site (Fkh250Con*). Although Hoxc6NΔ91 shows reduced binding in this assay, it is as effective as wildtype Hoxc6 in inducing LMC fates at thoracic levels (Figure 9). Equivalent molar amounts of Hox protein (2 pmol) were used in each assay.

To further define regions in Hoxc6 that contribute to its in vivo specificities we generated and characterized a series of HA-tagged deletion constructs and point mutations in Hoxc6. To discriminate between activities that influence DNA binding from those that affect target gene regulation, we tested the capacity of mutant derivatives to bind Hox recognition elements. To accomplish this we first needed to identify cognate sequences that are bound by Hoxc6 in vivo. Because *Foxp1* is regulated by Hox proteins we searched for potential Hox sites within the *Foxp1* locus. In silico analysis using the Vista enhancer browser [41] suggested a potential Hox-dependent enhancer upstream of *Foxp1* transcription start site. This enhancer (*Foxp1/hs1149*) is highly conserved amongst vertebrates and drives high levels of expression at limb levels of the spinal cord, and lower levels thoracically (Figure 7A). To test whether this element is regulated by *Hox* genes in vivo we bred a *Foxp1/hs1149::LacZ* line to *Hoxc9* mutants, in which all brachial *Hox* genes are derepressed at thoracic levels. This analysis revealed ectopic expression of *hs1149::LacZ* at thoracic levels (Figure 7A). These results identify a Hox-regulated element that targets the normal rostrocaudal domain where *Foxp1* expression is highest.

We next used gel mobility shift assays to determine if Hox proteins could bind the *hs1149* element. Since Pbx cofactors are generally necessary for high affinity Hox binding, we performed binding assays in the presence of Pbx3, a Pbx protein expressed by MNs. Scanning of the ∼1 kb *hs1149* enhancer identified a single potential binding site (TGAATTATCA), which generally conforms to the Hox/Pbx consensus [21]. We observed that Hoxc6 and Pbx3 cooperatively bound to this site in vitro (Figure 7B). To test whether Hox proteins bind this element in vivo we performed chromatin immunoprecipitation (ChIP) assays on chromatin prepared from e13.5 brachial and thoracic-level spinal cord using a Hoxc6 antibody. ChIP analysis revealed specific binding by Hoxc6 at brachial levels, but not at thoracic levels (Figure 7C), indicating the *hs1149* element is bound by Hoxc6 in vivo.

Using gel mobility shift assays we then tested the ability of Hoxc6 mutant derivatives to bind the *hs1149* element, as well as an optimized Hox consensus binding site (Fkh250con*) [22], [42]. We first tested the binding properties of mutant derivatives that would in principle preserve DNA recognition (i.e. retain the homeodomain [HD] and Pbx interaction motif [YPWM]) but might influence Hoxc6 activities at target sites. We tested the binding of two large N-terminal deletions, Hoxc6NΔ91 and Hoxc6NΔ111, finding that both deletions cooperatively bound with Pbx3 to *Foxp1*/*hs1149* and Fkh250con*, although with slightly reduced affinity (Figure 7D). In contrast a mutation of the conserved Pbx interaction motif (YPWM->AAAM) failed to display cooperative binding of Hoxc6 and Pbx3 to *Foxp1/hs1149* or Fkh250con* (Figure 7D).

### Analysis of motifs required for the diverse activities of Hoxc6

**Figure 8:**
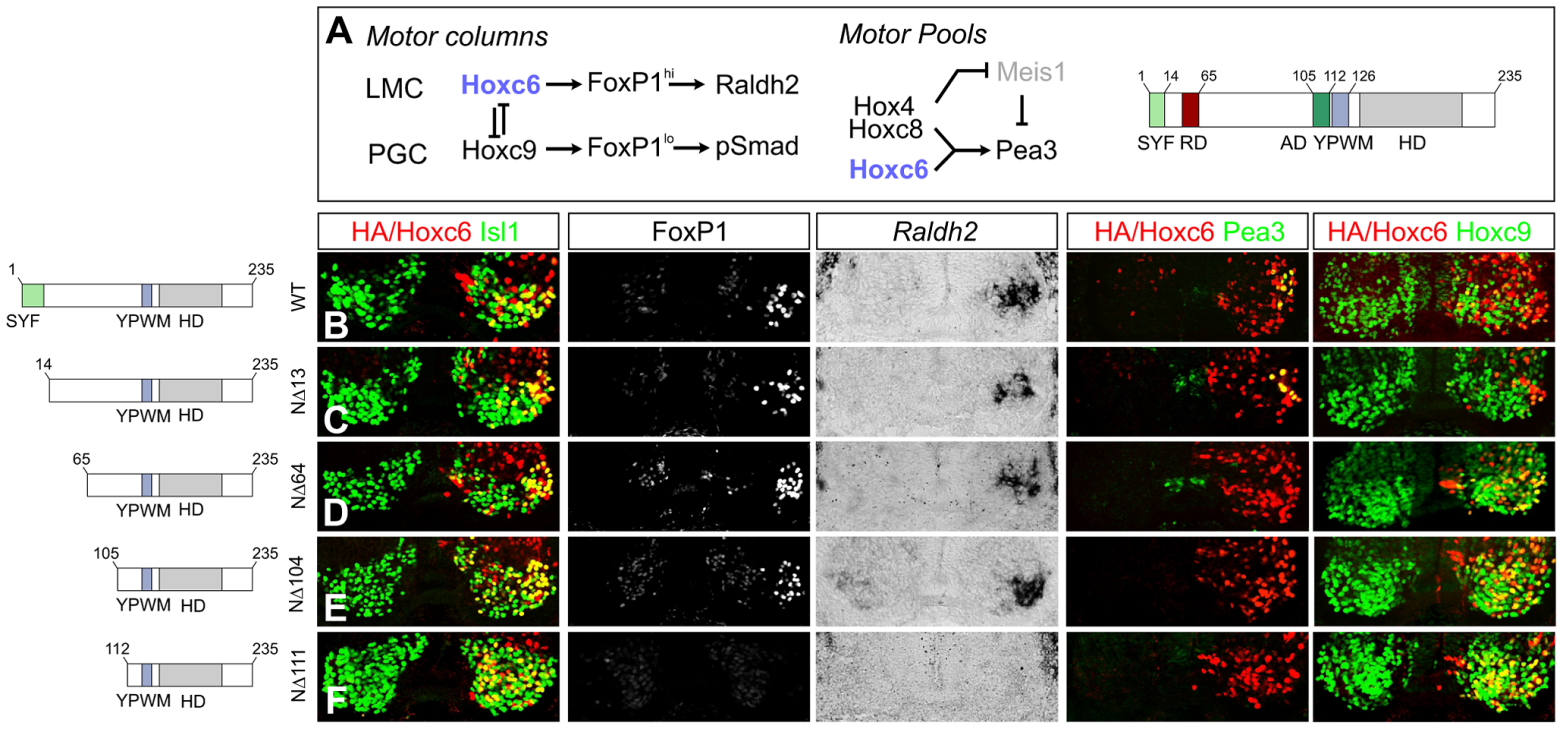
Specificity modules within the Hoxc6 protein. (A) Model of the transcriptional network controlling MN subtype identities at brachial and thoracic levels of the spinal cord. Hoxc6 promotes LMC fate through induction of high levels of FoxP1 and Raldh2 expression and restricts *Hoxc9*. Within the LMC a network of Hox4–Hox8 proteins controls pool fates. The network specifying the Pea3 pool is shown. Hoxc6 repression (RD) and activation domains (AD) are indicated. (B–F) Effects of expression of various Hoxc6 mutant derivatives on LMC specification at thoracic levels. Hoxc6 expression was monitored using either an HA antibody or an antibody against Hoxc6. Electroporated MNs are identified by co-staining with Isl1/2. (B) Hoxc6 activates *Foxp1*, *Raldh2*, *Pea3* and represses *Hoxc9*. (C) Deletion of the highly conserved “SYF” motif (Hoxc6NΔ13) does not affect Hoxc6 activity in MNs. (D–E) Hoxc6NΔ64 and Hoxc6NΔ104 induce LMC fate but fail to induce *Pea3* or repress *Hoxc9*. (F) Hoxc6NΔ111 fails to induce *Foxp1* and *Raldh2* or repress *Hoxc9*.

To test the in vivo activities of Hoxc6 mutant derivatives, we assessed their ability to influence MN differentiation at thoracic levels, using chick electroporation. To ensure that epitope tagged proteins were stable and that expression levels were similar amongst mutant derivatives, we monitored nuclear HA localization in electroporated cells and expressed mutant proteins at levels that were qualitatively similar to a HA-tagged wildtype Hoxc6 construct (Figure 8B). Expression of the large N-terminal deletion (Hoxc6NΔ111) at thoracic levels was inefficient in promoting *Foxp1*, *Raldh2*, *Pea3*, and repressing Hoxc9 expression (Figure 8F), indicating that the N-terminus is essential for Hoxc6 actions, independent of its ability to bind DNA.

We next tested the activities of additional deletion constructs within the N-terminus. Deletion of the first 13 amino acids, containing the highly conserved “SYF” motif present in the N-terminus of many Hox proteins, did not affect the ability of Hoxc6 to induce *Foxp1*, *Raldh2*, *Pea3* or repress *Hoxc9* at thoracic levels (Figure 8C). Further deletion of the N-terminal 64 amino acids (Hoxc6NΔ64) abrogated the capacity of Hoxc6 to repress *Hoxc9*, as neurons co-expressed both the deletion mutant and Hoxc9 (Figure 8D). Notably, the Hoxc6NΔ64 induced *Foxp1* and *Raldh2* expression, although this derivative was unable to generate Pea3+ MNs (Figure 8D). Hoxc6 can thus program LMC fate, even in the presence of a Hox protein that normally promotes thoracic fates. Further analysis revealed a requirement for amino acids 105–111 to activate LMC genes, as deletions up to amino acid 104 continued to induce high *Foxp1* and *Raldh2* expression (Figure 8E, 8F). These results indicate that the Hoxc6 N-terminus contains modular domains that are essential for diverse aspects of Hoxc6 function.

### Two distinct Hox-dependent mechanisms mediate LMC specification

**Figure 9:**
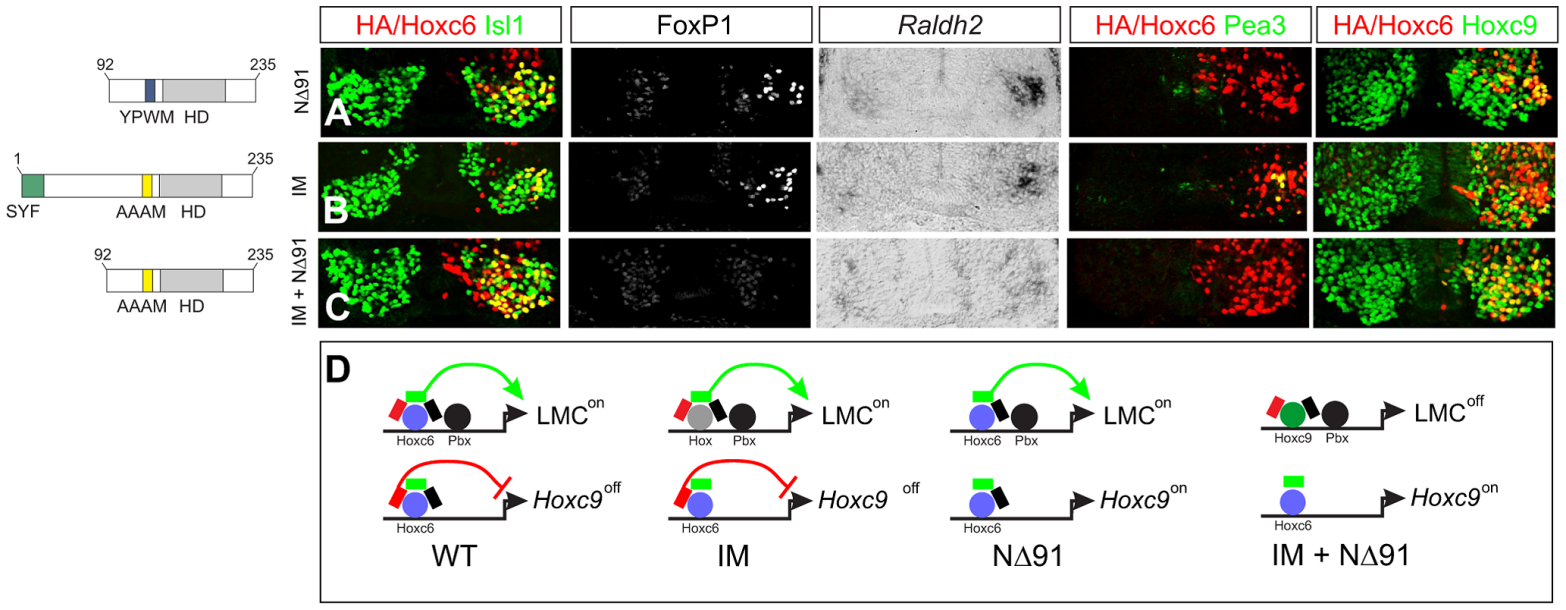
Pbx-dependent and -independent strategies for generating LMC neurons. (A) Expression of Hoxc6NΔ91 at thoracic levels activates *Foxp1* and *Raldh2*, but fails to induce *Pea3* or repress *Hoxc9*. (B) Expression of Hoxc6IM (YPWM->AAAM mutation) at thoracic levels activates *Foxp1*, *Raldh2*, *Pea3* and represses *Hoxc9*, similar to wildtype Hoxc6. The ability of Hoxc6IM to activate Pea3 could reflect a Pbx-independent program that specifies the Pea3+ pool. (C) Expression of Hoxc6NΔ91+IM fails to repress *Hoxc9* or induce LMC identity. (D) Interpretation of results. Hoxc6 normally induces LMC-specific genes in a Pbx-dependent manner, and contributes to the exclusion of *Hoxc9*. When the Pbx interaction domain is mutated, *Hoxc9* is still repressed. As Hoxc9 normally acts to dampen *Foxp1* expression, the absence of Hoxc9 allows Hox proteins resident to the thoracic spinal cord to induce *Foxp1* and activate the LMC program. When both the Pbx interaction motif and *Hoxc9* repression domain are deleted, *Hoxc9* is expressed, ensuring *Foxp1* is not activated, and preventing LMC specification.

Because Hox proteins often require cooperative interactions with Pbx proteins to bind DNA, we also examined the consequences of mutating the YPWM motif (Hoxc6IM: YPWM->AAAM). Surprisingly expression of Hoxc6IM did not alter the capacity of Hoxc6 to repress *Hoxc9* or to induce high *Foxp1* levels and *Raldh2* at thoracic levels (Figure 9B). Because Hoxc6IM retains the capacity to repress *Hoxc9*, and deletion of *Hoxc9* in mice leads to the derepression of brachial *Hox* genes at thoracic levels [26], we considered the possibility that expression of Hoxc6IM causes ectopic *Hox4–Hox8* gene expression, and that these in turn activate the LMC program. However, we did not observe ectopic brachial *Hox* gene expression after expression of Hoxc6IM at thoracic levels (data not shown), possibly because Hoxc9-mediated repression is established prior to the time Hoxc6IM is expressed. These observations suggest that either Pbx interactions are dispensable for LMC specification, or that the LMC neurons produced under these conditions reflect the actions of Hox proteins resident to the thoracic spinal cord whose functions are unmasked through suppression of *Hoxc9*.

To test this idea we generated a Hoxc6 derivative in which both the *Hoxc9* repression domain (NΔ91) and YPWM motif were mutated, but retained the intrinsic activation domain (aa 105–111). While Hoxc6 NΔ91 cooperatively bound with Pbx3 to the *hs1149* element and Fkh250con* and activated LMC genes (Figure 9A and Figure 7D), the combined NΔ91/IM mutant failed to bind or reprogram thoracic MNs to an LMC fate (Figure 9C and Figure 7D). Thus only through removal of both the *Hoxc9* repression domain and Pbx interaction motif is the ability of Hoxc6 to promote LMC fate lost. These results suggest that Hoxc6 can promote LMC identity through two distinct mechanisms 1) Pbx-independent restriction of *Hoxc9* and 2) Pbx-dependent interactions with *Foxp1*, activities that are separable (Figure 9D). Together these data indicate Hoxc6 contains specific motifs required for LMC induction, cross-repressive interactions, and pool specification, with LMC induction mediated by redundant activities of Hox cross repression and cooperative interactions with Pbx proteins.

## Discussion

The specification of LMC neurons is a critical step in the genetic pathways controlling innervation of limb musculature and is central in the emergence of locomotor circuitry [2], [9]. At brachial and lumbar levels of the spinal cord expression of Hox6 and Hox10 proteins are closely aligned with the position in which LMC neurons are generated. However the specific requirements for *Hox6* gene function during LMC specification were not known. In this study we have found that *Hox6* genes are required for the normal generation of LMC neurons and the specification of the Pea3+ pool. In the absence of *Hox6* genes, LMC identity appears to be preserved through the actions of an unexpectedly diverse set of Hox proteins expressed by brachial MNs. Our findings indicate that while multiple Hox-dependent strategies are deployed to establish the columnar identity of limb-innervating MNs, more restricted activities emerge at the level of pool specification. The convergent and restricted actions of Hox proteins in LMC neurons are likely to be a general feature of Hox function in both vertebrate and invertebrate systems.

### Hox determinants of motor neuron columnar identity and position

While experiments in chick have implicated *Hox6* gene function in the specification of brachial LMC neurons, we find in mice lacking all *Hox6* activity that LMC neurons are still generated, although significantly reduced in numbers. In the absence of *Hox6* genes the forelimb LMC program appears to be maintained by a large set of *Hox5–Hox8* genes, which likely act by promoting high levels of *Foxp1* expression in MNs. This idea is supported by the observations that multiple *Hox* paralogs are expressed by brachial MNs, and that Hox5–Hox8 proteins can convert PGC neurons to an LMC identity when expressed at thoracic levels. Thus a diverse group of Hox proteins promote columnar identities, through regulating a common set of genes capable of interpreting Hox inputs from several paralog groups. Similar redundant activities amongst diverse Hox paralogs have been demonstrated during haematopoiesis [43], suggesting that in certain cellular contexts key target effectors may permissively engage any Hox protein present. The non-selective Hox inputs to LMC-restricted genes may further explain the ability of a dominant repressor form of Hoxc6 to inhibit LMC specification [11], by superseding the compensatory actions of Hox5–Hox8 proteins.

Why then do so many *Hox* genes contribute to LMC identity? The simplest explanation is that it represents an extreme example of functional redundancy, ensuring that spurious *Hox* mutations do not lead to devastating consequences for the organism. Alternatively, it may reflect a strategy employed to allow individual Hox proteins to exert distinct functions in MN pool specification, with redundant Hox factors serving the role of maintaining LMC identity over the course of MN differentiation. The Hox protein most closely aligned with the brachial LMC, Hoxc6, displays a dynamic expression pattern during LMC differentiation, with many MNs attenuating Hoxc6 expression shortly after columnar identity is established. This pattern may allow for MN pool subtypes that might otherwise be inhibited by the presence of Hoxc6 to retain an LMC fate, while simultaneously acquiring a unique pool identity.

The logic of the limb-level Hox network in MNs contrasts with the columnar program at thoracic levels of the spinal cord, which is largely mediated through the actions of a single *Hox* gene, *Hoxc9* [26]. In addition to maintaining low levels of FoxP1 in PGC neurons, an essential function of Hoxc9 is to restrict expression of *Hox4–Hox8* genes to brachial levels. Thus while brachial MNs express many Hox factors that appear to act on common LMC targets, thoracic MNs express relatively few *Hox* genes, one of which operates to restrict expression of multiple *Hox* genes, in an apparently selective manner. The distinct transcriptional strategies employed at these two levels of the spinal cord are therefore uniquely geared towards either confining or expanding the diversity of MN subtypes, in accordance with the relative complexity of the target tissues they innervate.

### Selective actions of Hox proteins in motor neuron pool specification

Although individual *Hox* genes are largely dispensable for early programs of LMC differentiation, they are critical in the specification of motor neuron pools. While *Hoxc6* mutants display no significant LMC losses in caudal brachial regions, the MN pool defined by Pea3 is markedly reduced in size. Presumably the LMC is preserved in this region due to compensatory actions of Hoxa7 and Hoxc8, which maintain high FoxP1 levels but are apparently insufficient to promote the normal pattern of Pea3 expression. This differential effect on columnar and pool specification likely reflects distinct Hox specificities in the regulation of target effectors, with LMC determinants like *Foxp1* and *Raldh2* integrating multiple Hox inputs, and pool specific genes relying on specific *Hox* gene activities. This mechanism mirrors the “semi-paralog specific” and “paralog-specific” activities of Hox proteins observed in *Drosophila* [17]. Our data indicate that these distinct modes of Hox-dependent gene regulation operate when neurons are confronted by multiple Hox proteins within the same cell.

Previous studies in chick indicate that the specification of the MN pools defined by Pea3 and Scip expression involve the combinatorial interactions between multiple Hox proteins and their cofactors, including Hoxc4, Hoxc6, Hoxc8 and Meis1 [10]. It is somewhat surprising that expression of Hoxc6 is sufficient to direct the specification of the Pea3 pool independent of these factors. This finding however appears to reflect how the network operates within the LMC. A primary function of Hoxc8 and Hoxc4 is to exclude Meis1 from a subset of MNs, with Hoxc6 acting to promote Pea3 expression within the context of a Meis1− LMC neuron [10]. Thoracic MNs contain an endogenous subpopulation that lacks Meis1 (J. Dasen unpublished observation), allowing Hoxc6 to promote a Pea3+ pool identity. Thus while individual Hox proteins can promote pool fates, these activities are normally constrained by the actions of other Hox proteins in the network, and can only operate in the context of a MN that has been programmed to an LMC identity.

### Regulating Hox activities during motor neuron differentiation

While regions outside the homeodomain that contribute to the specificities of Hox proteins in *Drosophila* have been well documented [44]–[47], few studies have defined functional motifs in the vertebrate nervous system. Our analysis of regulatory domains within the Hoxc6 protein provides insight into the mechanisms through which Hox proteins contribute to MN columnar and pool identities. We find that Hoxc6 contains N-terminal motifs that are critical for deployment of LMC programs as well as cross-repressive functions, independent of its core DNA binding domain. Surprisingly either of these activities is sufficient to promote LMC identity. Removal of the *Hoxc9* repression domain does not affect Hoxc6's capacity to generate LMC neurons at thoracic levels, even in the presence of an inhibitory Hox program. The underlying mechanism for this phenomenon likely reflects how Hox proteins regulate LMC-targets, such as *Foxp1*. *Foxp1* is induced at low levels by Hoxc9 in thoracic MNs, and thus appears to be equally capable of receiving inputs from both limb and thoracic-level Hox proteins. Forced expression of LMC Hox determinants (Hox5–Hox8 paralogs) presumably acts by overriding the dampening influence of Hoxc9, perhaps by competing for binding sites. This idea is supported by the observation that Hox5–Hox8 proteins can induce aspects of LMC differentiation without suppressing *Hoxc9*. During normal development this process is likely played out in two distinct contexts: at the border between brachial and thoracic spinal cord, where many LMC MNs express low levels of Hoxc9 [26], and within the LMC where FoxP1 is maintained by pools of MNs which have lost Hoxc6 expression. Competitive and compensatory interactions between Hox proteins at genomic targets therefore may be more generally utilized in MNs both as means to generate diversity and to allow for certain gene programs to be maintained in the face of dynamic transcription factor profiles.

Our findings indicate that suppression of *Hoxc9* is a condition sufficient to promote LMC fates even in the absence of a functional LMC Hox protein. When the Pbx interaction motif is deleted from Hoxc6, *Hoxc9* is still repressed and LMC neurons are generated, although Hoxc6 presumably will no longer have a direct impact on activating the LMC program. One plausible explanation for this result is that the suppression of *Hoxc9* unmasks the activities of Hox proteins that are endogenous to the thoracic spinal cord that have the capacity to activate high levels of *Foxp1* (Figure 9D). While the identity of these Hox proteins is unclear, they may include more broadly expressed *Hox* genes (e.g. Hoxd4 [48]) or the activities of “brachial” *Hox* genes, such as *Hoxa7* and *Hoxc8*, which are expressed at low levels in chick thoracic spinal cord [10]. Thus we favor a model in which in the absence of Pbx interactions, the repressive influence of Hoxc6 on *Hoxc9* is retained, and LMC neurons are generated through a Pbx-dependent mechanism that utilizes Hox proteins resident to thoracic levels of the spinal cord (Figure 9). This idea is consistent with studies in flies indicating that Hox proteins repress targets in the absence of Pbx interactions where they bind as monomers [49]. In addition our observations may also account for the finding that when similar Pbx-interaction mutations are generated in the lumbar LMC-determinant Hoxd10, LMC fates are induced when expressed at thoracic levels [50].

Collectively these studies add to emerging evidence that the specific actions of Hox proteins are defined through peptide modules that shape both the selection of DNA targets, and gate the activities of Hox proteins once bound to a site [17], [38], [39], [44], [51]. While the precise mechanisms that determine how Hox proteins deploy their restricted actions in MNs are unresolved, they likely rely on gene-specific interactions of Hox proteins with other transcription factors or cosignaling partners [17]. Our studies indicate that the subfunctionalization of Hoxc6, and Hox proteins in general, into motifs that confer the activation and repression at multiple gene targets can expand the repertoire of Hox function even within a single cell, allowing them to execute their multifaceted roles during cellular differentiation.

## Materials and Methods

### Mouse genetics

The *Hox6* mutant strains [33], and the *Hb9::GFP* line [36], have been described previously.

### *In ovo* chick embryo electroporation

Electroporation was performed on stage 12 to 16 chick embryos as described [11]. Results for each experiment are representative of at least three electroporated embryos from three or more independent experiments in which the electroporation efficiency in MNs was >60%. The amount of pCAGGs plasmid DNA in each electroporation was titrated to achieve levels of expression qualitatively similar to endogenous expression levels, typically in the range of 100–300 ng/µl, using pBKS as carrier DNA (1.8–2 µg/µl). Electroporations of mutant and chimera Hox derivatives were optimized to ensure qualitatively similar levels of expression to endogenous Hox levels by comparing HA-wildtype Hoxc6 to non-tagged Hoxc6, and comparing HA-tagged Hoxc6 to HA-tagged mutant derivatives. Stability of expression of these constructs was determined by the presence of nuclear HA staining.

### ChIP assays

Chromatin immunoprecipitation was performed as previously described [26]. Briefly, brachial and thoracic spinal cords were dissected from e13.5 mouse embryos. Tissues were homogenized in 1.1% formaldehyde, chromatin was extracted and fragmented to 500–1000 bp by sonication and chromatin extracts were subjected to immunoprecipitation with either specific antibodies or species-matched IgGs. Antibodies used were goat anti-mouse Hoxc6 (Santa Cruz) or rabbit ant-mouse Hoxc6 (Abcam). Genomic regions were amplified using Sybr Green PCR Master Mix (Applied Biosystems) and detected with Mx 3005P real-time PCR apparatus (Stratagene). Fold enrichment was calculated over IgG using the ΔΔCt method: fold enrichment = 2^−(ΔΔCt)^, where ΔΔCt = (Ct_IP_−Ct_Input_)−(Ct_IgG_−Ct_Input_).

### Recombinant protein induction and purification

pET-14b plasmids carrying His-tagged constructs were used to transform BL21 pLys bacterial strain and protein expression was induced by 0.5 mM IPTG at room temperature overnight. Bacteria were lysed under native conditions in lysis buffer (50 mM Tris pH 7.5 and 100 mM NaCl) followed by sonication. The supernatant was incubated with Ni-NTA agarose beads at 4°C for 1.5 hours and washed three times in buffer containing 50 mM Tris pH 7.5, 300 mM NaCl, 20 mM imidazole and 0.5% Igepal CA-630. Recombinant proteins were eluted in 50 mM Tris pH 7.5, 300 mM NaCl, 250 mM imidazole and dialyzed overnight in 50 mM Tris-HCl pH 8 and 150 mM NaCl.

### Gel shift assays

Oligonucleotides containing putative Hox binding sites were annealed to an IRDye-800 labeled linker (IRD800-AGCTGTGGGACGAGG). Double stranded probes were synthesized using Klenow DNA polymerase. Binding between recombinant proteins and DNA probes was performed in binding buffer containing 50 mM Tris-HCl pH 7.5, 250 mM NaCl, 5 mM MgCl2, 20% Glycerol, 2.5 mM DTT, 2.5 mM EDTA pH 8, 250 ng/µL poly dIdC and 0.1% BSA for 20 minutes at room temperature. For each binding assay equivalent molar amounts (0.5–2 pmol/reaction) of recombinant protein were used. Binding reactions were resolved on a non-denaturing acrylamide gel and the IRDye-800 was detected using the Odyssey system (Li-Cor).

### *In situ* hybridization and immunohistochemistry

*In situ* hybridization and immunohistochemistry were performed on 16 µm cryostat sections as described [52]. Whole-mount antibody staining was performed as described [14] and GFP-labeled motor axons were visualized in projections of confocal Z-stacks (500–1000 µm). Antibodies against Hox proteins, LIM HD proteins, and other proteins were generated as described [10], [27], [28], [52]. Retrograde labeling of MNs was performed as described [28].

### Ethics statement

Procedures involving animals abide by the NYUMC policy on the care and use of laboratory animals. Experiments involving animals are not conducted unless approved by the Institutional Animal Care and Use Committee (IACUC). We do not work with a species or procedure, including euthanasia, with which I and those members of my research staff involved in this project are not experienced, without first seeking the advice and instruction of a veterinarian from the Division of Laboratory Animal Resources, consult the Division of Laboratory Animal Resources as circumstances require. To the best of my knowledge, the research does not unnecessarily duplicate previous research with respect to the use of laboratory animals. We comply with all requests for data as may be required by governmental and institutional guidelines. We seek the approval of the Institutional Animal Care and Use Committee on all procedures which involve laboratory animals.

## Supporting Information

Figure S1 Analysis of MN columnar specification in *Hoxa6/c6* mutants at e11.5. (A) Increase in the number of HMC neurons at rostral brachial levels in *Hoxa6/Hoxc6* mutants at e11.5. Serial sections from rostral to caudal levels of the LMC are shown left to right. HMC neurons are identified by Hb9+Isl1/2 coexpression, indicated in cyan. (B, C) Normal expression of Hoxc4 and Hoxc8 in *Hox6* mutants at e11.5. Serial sections along the rostrocaudal axis showing normal expression of Hoxc8 and Hoxc4 in FoxP1+ LMC neurons. *HoxA* genes are also expressed normally in *Hox6* mutants (data not shown).
(PDF)

Figure S2 *Hoxc9* is not derepressed at brachial levels in *Hoxa6/Hoxc6* mutants. Serial sections at caudal brachial levels showing that Hoxc9 is normally restricted from FoxP1+ LMC neurons in *Hoxc6* and *Hoxa6/Hoxc6* mutants. At these levels Hoxc9 is normally expressed in neurons located dorsal to the LMC. Rostral to caudal is shown left to right.
(PDF)

Figure S3 Analysis of LMC specification in *Hox6* mutants. (A) Total number of FoxP1+ LMC neurons in the brachial spinal cord of various *Hox6* mutant allele combinations. (B) Levels of FoxP1 protein expression are reduced in rostral brachial regions in *Hoxa6/Hoxc6* mutants. Levels were determined by measuring the pixel intensities of FoxP1 nuclear staining. (C, D) Decrease in the number of FoxP1+ LMC neurons at brachial levels in *Hoxa6/Hoxc6* mutants at e10.5 and e11.5. Images show serial sections along the rostrocaudal axis from left to right. Loss of FoxP1 is prominent at rostral brachial levels (Hoxa5/Hoxc5+ region) of the spinal cord. Approximate position of the Hox5/Hoxc8 boundary is indicated.
(PDF)

Figure S4 Analysis of LMC specification in *Hox6* triple mutants. In mice lacking all three *Hox6* genes (*Hoxa6*, *Hoxb6*, *Hoxc6*) LMC neurons are still generated in caudal brachial spinal cord, as assessed by FoxP1 and Raldh2 expression. In rostral brachial spinal cord, there is an additional loss in LMC neurons in triple mutants when compared to *Hoxb6/c6* double mutants, but essentially phenocopies the LMC loss in *Hoxa6/c6* double mutants (See Figure 2).
(PDF)

Figure S5 Efficiency of LMC induction by Hox4–Hox8 proteins. (A) Examples of Hox electroporations in chick showing similar levels of protein expression to endogenous brachial levels. (B) Quantification of mean pixel intensities of Hox staining in n>40 nuclei of electroporated neurons at brachial and thoracic levels. (C) Quantification of the percentage of electroporated MNs (defined by Isl1/2 expression) that express high levels of FoxP1 at thoracic levels after misexpression of the indicated *Hox* gene. Error bars show s.e.m.
(PDF)

Figure S6 Motor neuron pool defects in *Hoxc6* mutants. (A–D) Additional examples of whole mount GFP staining showing defects in motor axon innervation of the cm muscle in *Hoxc6* mutants at e12.5 and e13.5. (E) Loss of Pea3+ and retention of Scip+ motor neuron pools at e11.5 in *Hox6* mutants. There is a marked decrease in the number of Pea3+ MNs at e11.5 in *Hoxa6/Hoxc6* mutants.
(PDF)

Figure S7 Analysis of tracer injections into the ulnar nerve in Hoxc6 mutants. (A) Summary of the position and distribution of the Pea3+ and Scip+ MN pools in the caudal half of the lateral motor column (cLMC). Relative positions of the pools in transverse sections are indicated for both control and *Hoxc6* mutants. (B) Summary of the distribution of labeled MNs after ulnar injection. In control mice only Scip+ MNs are labeled. In *Hoxc6* mutant mice Scip− MNs are labeled, the position of these labeled MNs extends rostrally, and overlaps with the position of the former Pea3+ MN pool. (C) Serial sections from rostral to caudal showing distribution of labeled MNs after ulnar injections in control and *Hoxc6* mutant mice.
(PDF)
