# Supplementary figures and images for: Genetic and Functional Modularity of Hox Activities in the Specification of Limb-Innervating Motor Neurons
Source: PLoS Genet. 2013 Jan 24;9(1):e1003184. doi: 10.1371/journal.pgen.1003184 (PMC3554521; doi:10.1371/journal.pgen.1003184)

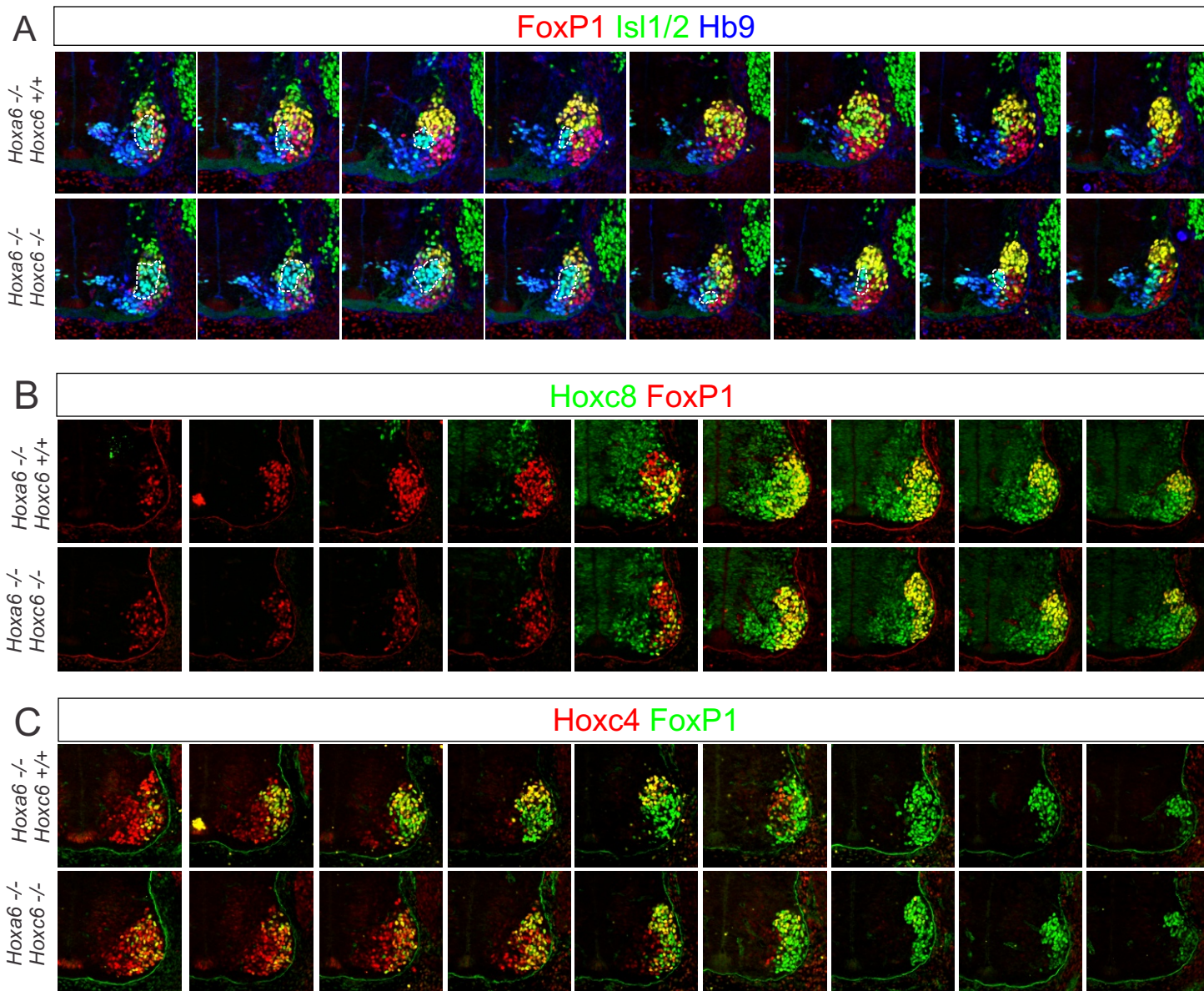

Supplement: Figure S1 — Analysis of MN columnar specification in Hoxa6/c6 mutants at e11.5. (A) Increase in the number of HMC neurons at rostral brachial levels in Hoxa6/Hoxc6 mutants at e11.5. Serial sections from rostral to caudal levels of the LMC are shown left to right. HMC neurons are identified by Hb9+Isl1/2 coexpression, indicated in cyan. (B, C) Normal expression of Hoxc4 and Hoxc8 in Hox6 mutants at e11.5. Serial sections along the rostrocaudal axis showing normal expression of Hoxc8 and Hoxc4 in FoxP1+ LMC neurons. HoxA genes are also expressed normally in Hox6 mutants (data not shown). (PDF) [file pgen.1003184.s001.pdf]

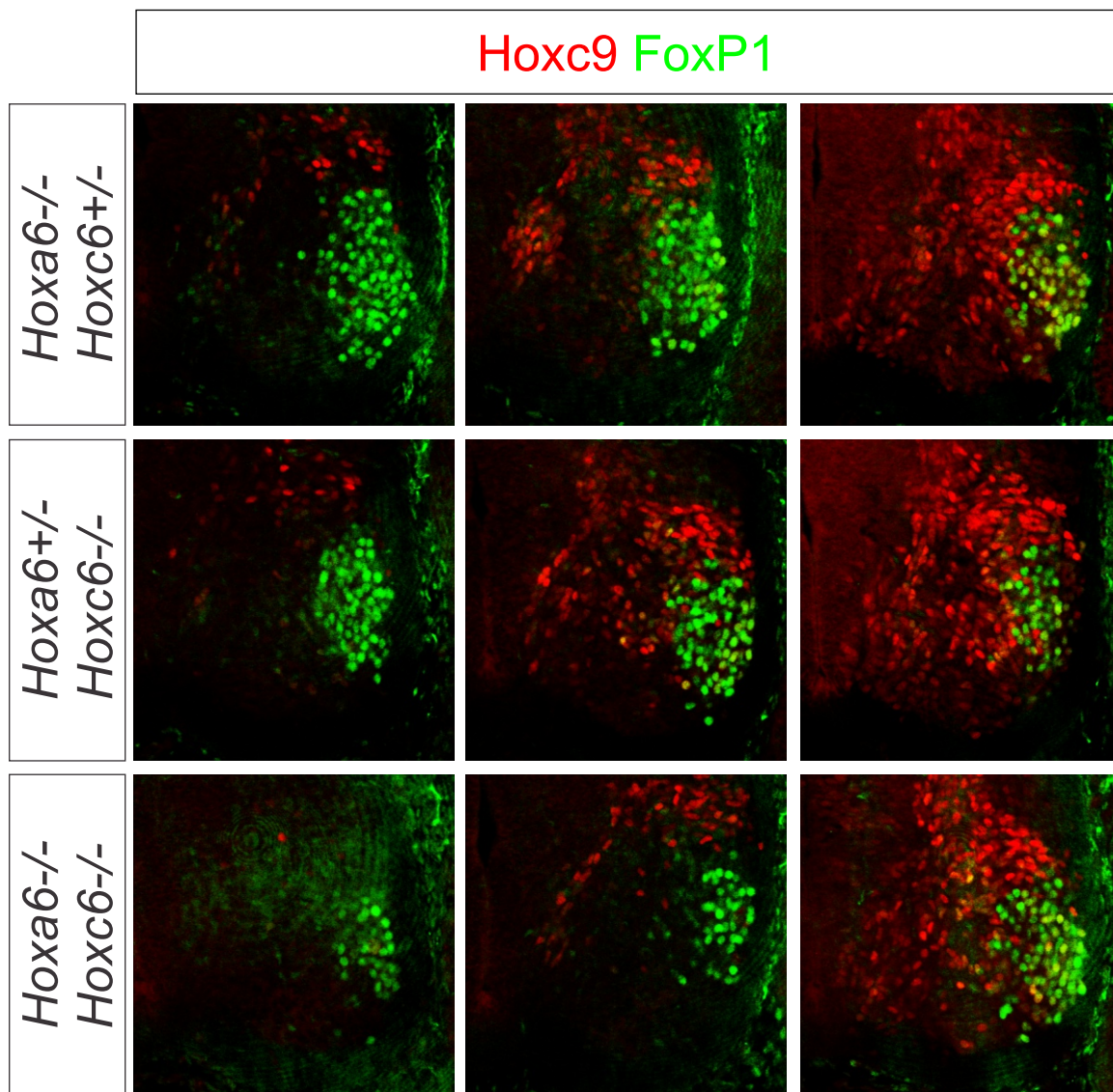

Supplement: Figure S2 — Hoxc9 is not derepressed at brachial levels in Hoxa6/Hoxc6 mutants. Serial sections at caudal brachial levels showing that Hoxc9 is normally restricted from FoxP1+ LMC neurons in Hoxc6 and Hoxa6/Hoxc6 mutants. At these levels Hoxc9 is normally expressed in neurons located dorsal to the LMC. Rostral to caudal is shown left to right. (PDF) [file pgen.1003184.s002.pdf]

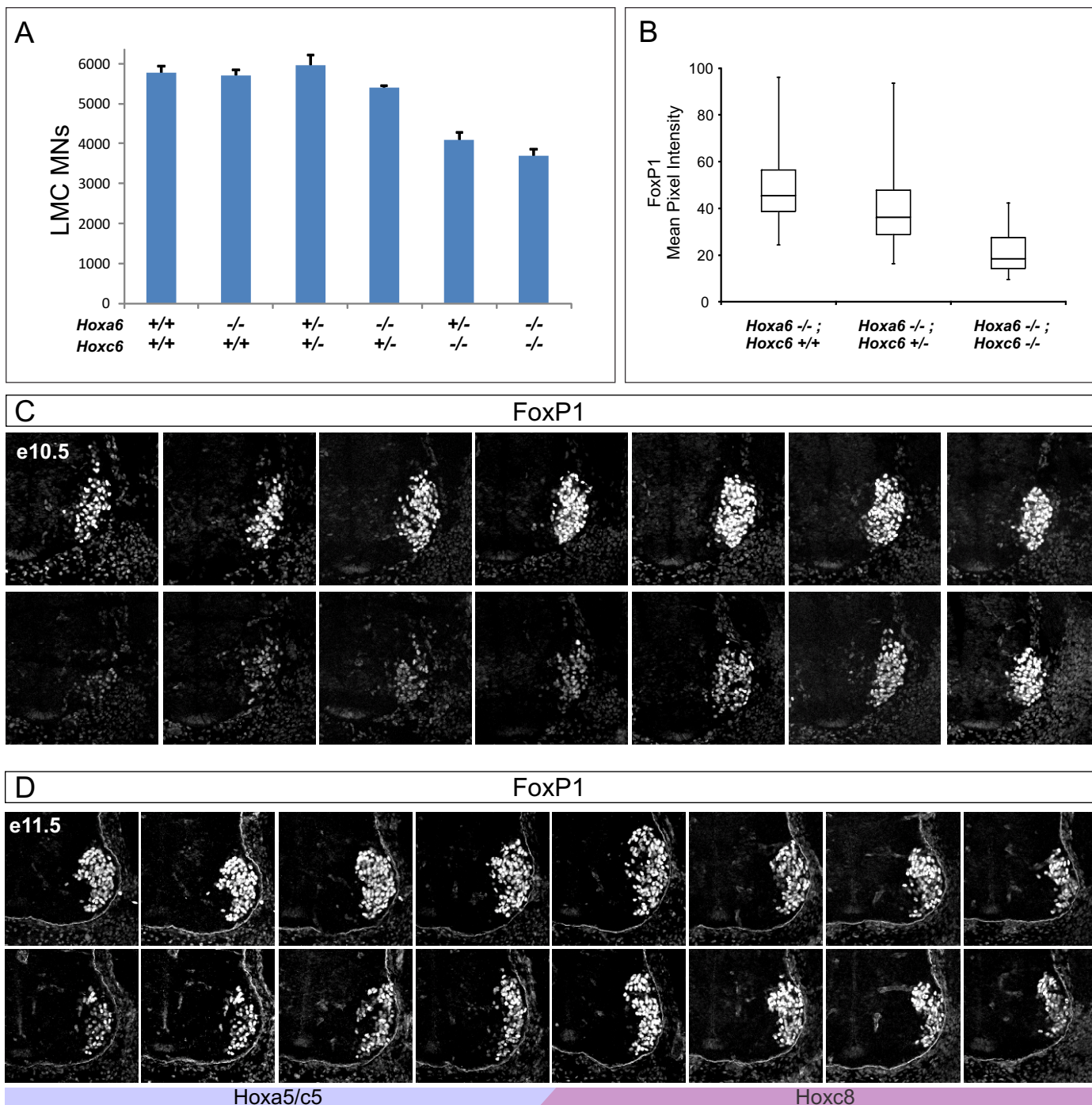

Supplement: Figure S3 — Analysis of LMC specification in Hox6 mutants. (A) Total number of FoxP1+ LMC neurons in the brachial spinal cord of various Hox6 mutant allele combinations. (B) Levels of FoxP1 protein expression are reduced in rostral brachial regions in Hoxa6/Hoxc6 mutants. Levels were determined by measuring the pixel intensities of FoxP1 nuclear staining. (C, D) Decrease in the number of FoxP1+ LMC neurons at brachial levels in Hoxa6/Hoxc6 mutants at e10.5 and e11.5. Images show serial sections along the rostrocaudal axis from left to right. Loss of FoxP1 is prominent at rostral brachial levels (Hoxa5/Hoxc5+ region) of the spinal cord. Approximate position of the Hox5/Hoxc8 boundary is indicated. (PDF) [file pgen.1003184.s003.pdf]

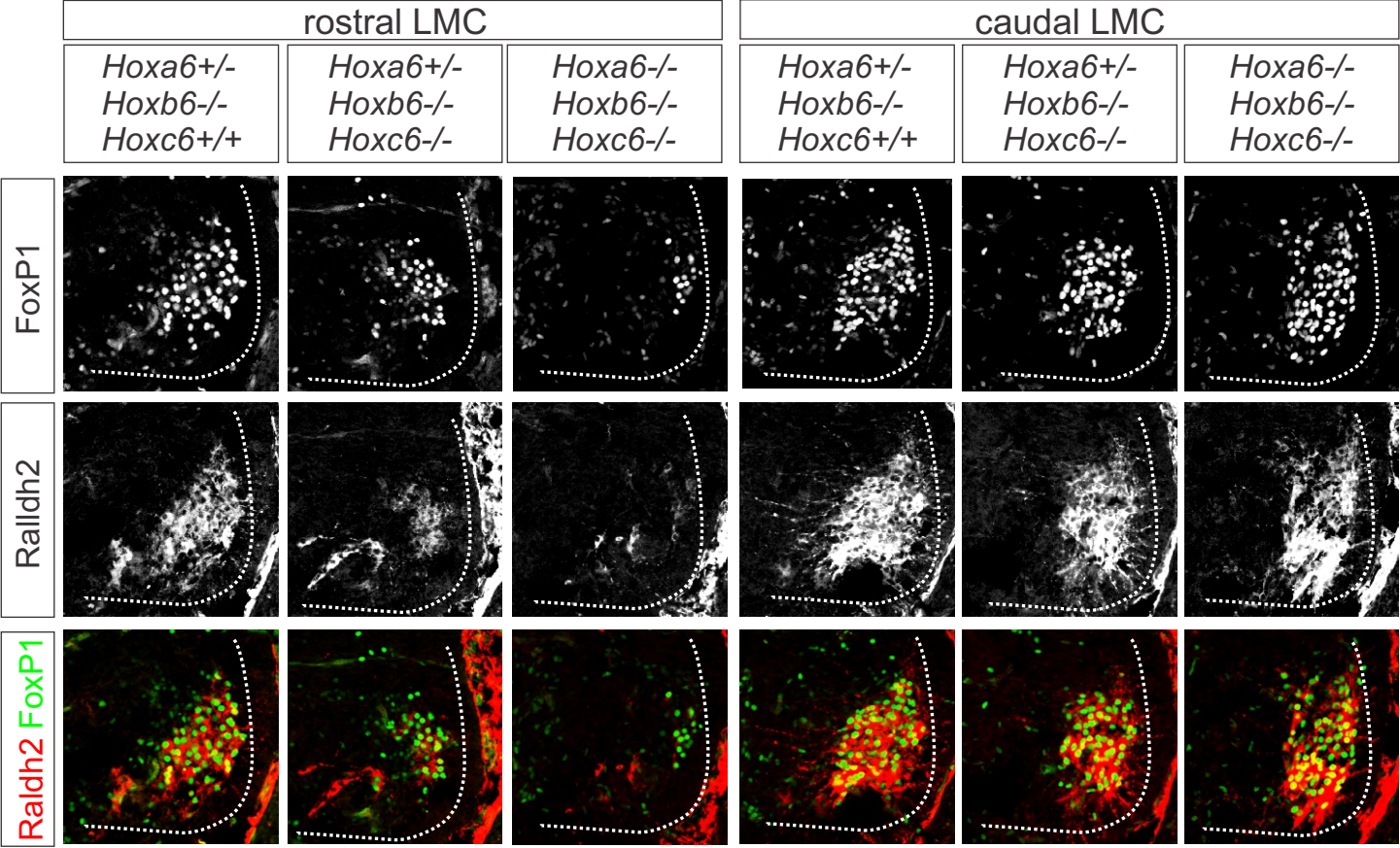

Supplement: Figure S4 — Analysis of LMC specification in Hox6 triple mutants. In mice lacking all three Hox6 genes (Hoxa6, Hoxb6, Hoxc6) LMC neurons are still generated in caudal brachial spinal cord, as assessed by FoxP1 and Raldh2 expression. In rostral brachial spinal cord, there is an additional loss in LMC neurons in triple mutants when compared to Hoxb6/c6 double mutants, but essentially phenocopies the LMC loss in Hoxa6/c6 double mutants (See Figure 2). (PDF) [file pgen.1003184.s004.pdf]

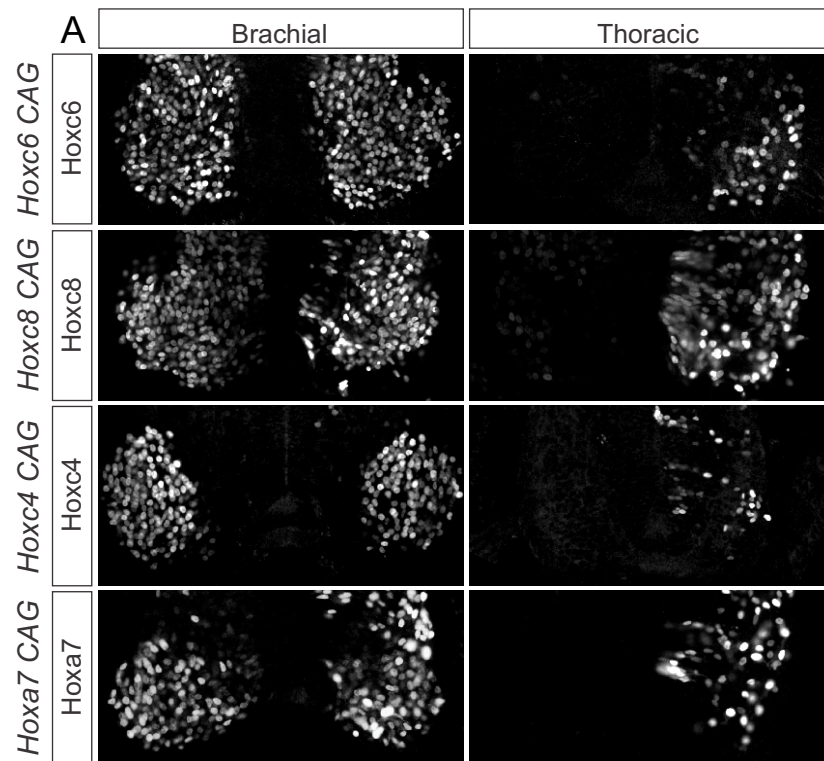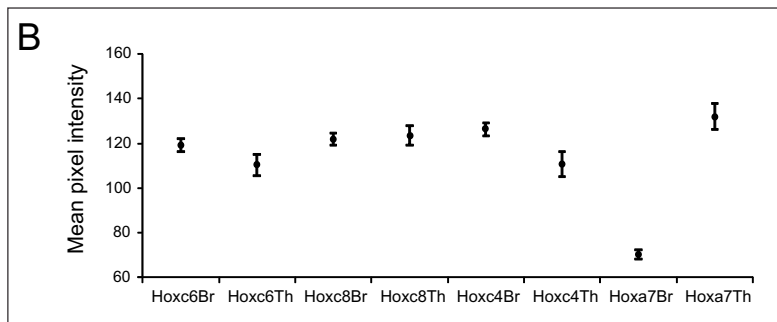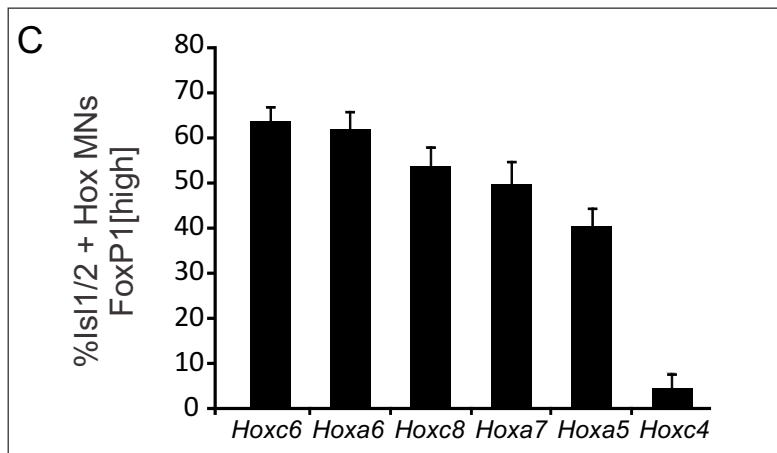

Supplement: Figure S5 — Efficiency of LMC induction by Hox4–Hox8 proteins. (A) Examples of Hox electroporations in chick showing similar levels of protein expression to endogenous brachial levels. (B) Quantification of mean pixel intensities of Hox staining in n>40 nuclei of electroporated neurons at brachial and thoracic levels. (C) Quantification of the percentage of electroporated MNs (defined by Isl1/2 expression) that express high levels of FoxP1 at thoracic levels after misexpression of the indicated Hox gene. Error bars show s.e.m. (PDF) [file pgen.1003184.s005.pdf]

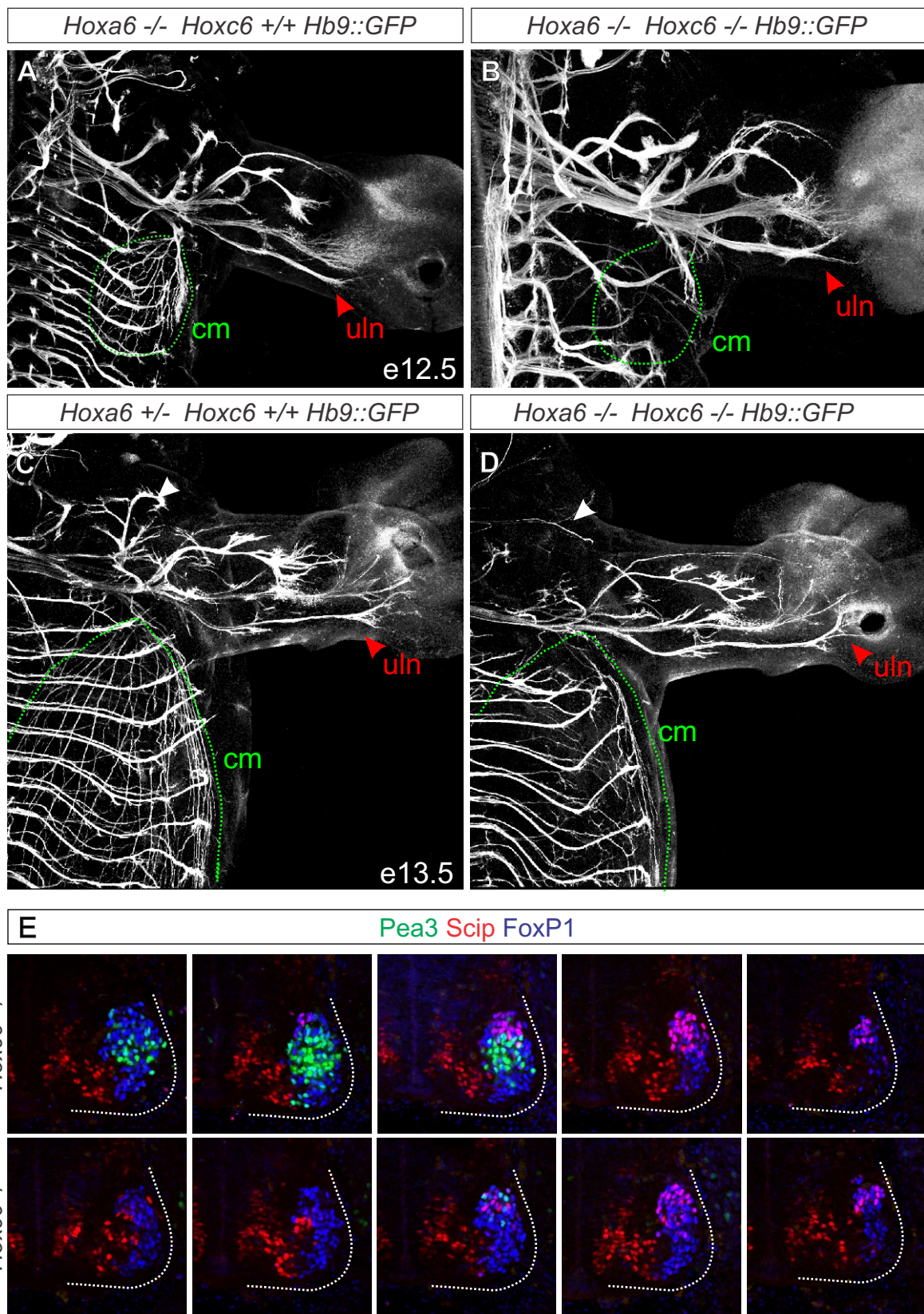

Supplement: Figure S6 — Motor neuron pool defects in Hoxc6 mutants. (A–D) Additional examples of whole mount GFP staining showing defects in motor axon innervation of the cm muscle in Hoxc6 mutants at e12.5 and e13.5. (E) Loss of Pea3+ and retention of Scip+ motor neuron pools at e11.5 in Hox6 mutants. There is a marked decrease in the number of Pea3+ MNs at e11.5 in Hoxa6/Hoxc6 mutants. (PDF) [file pgen.1003184.s006.pdf]

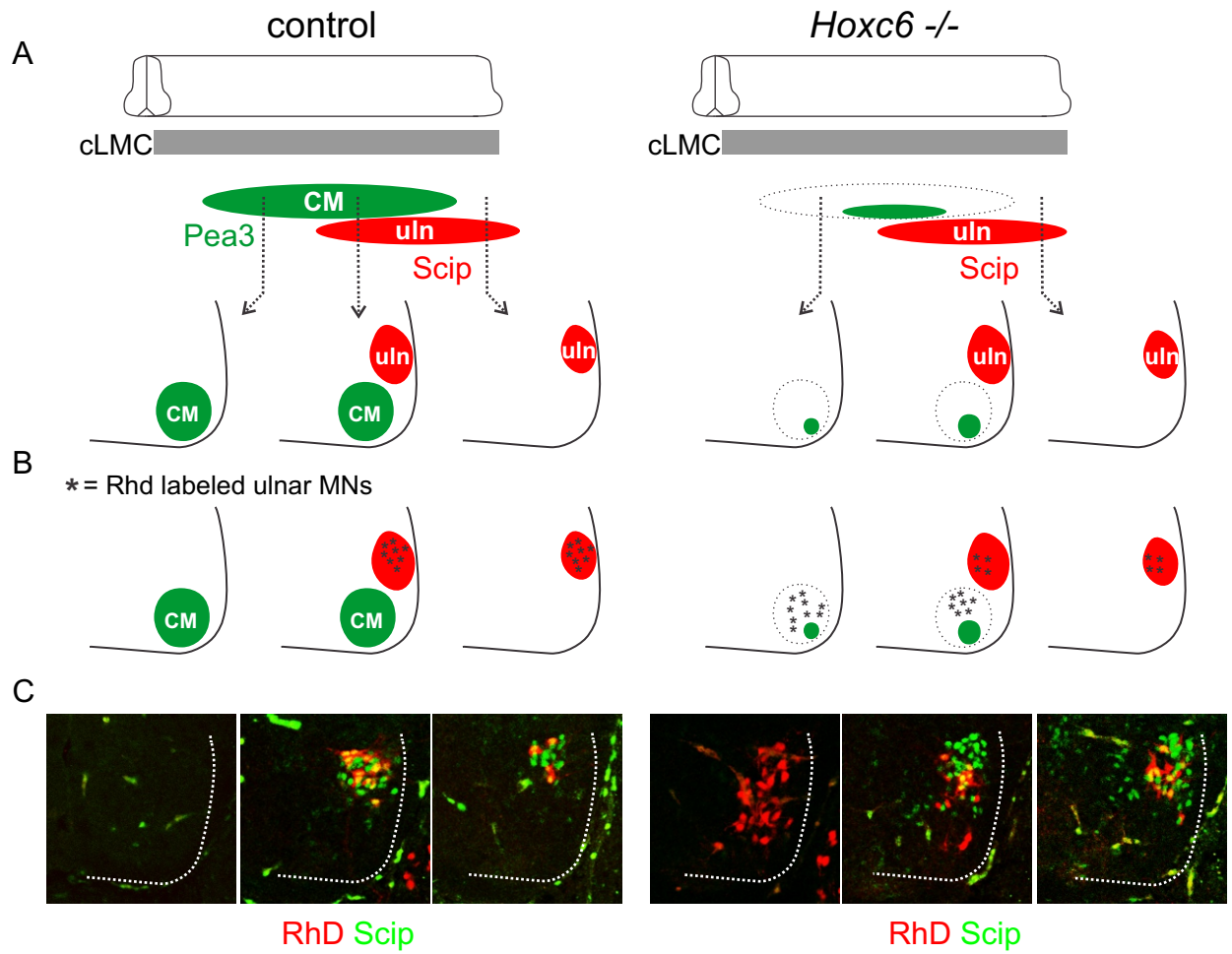

Supplement: Figure S7 — Analysis of tracer injections into the ulnar nerve in Hoxc6 mutants. (A) Summary of the position and distribution of the Pea3+ and Scip+ MN pools in the caudal half of the lateral motor column (cLMC). Relative positions of the pools in transverse sections are indicated for both control and Hoxc6 mutants. (B) Summary of the distribution of labeled MNs after ulnar injection. In control mice only Scip+ MNs are labeled. In Hoxc6 mutant mice Scip− MNs are labeled, the position of these labeled MNs extends rostrally, and overlaps with the position of the former Pea3+ MN pool. (C) Serial sections from rostral to caudal showing distribution of labeled MNs after ulnar injections in control and Hoxc6 mutant mice. (PDF) [file pgen.1003184.s007.pdf]
